# Machine Learning Prediction of Resistance to Subinhibitory Antimicrobial Concentrations from Escherichia coli Genomes

**DOI:** 10.1128/mSystems.00346-21

**Published:** 2021-08-24

**Authors:** Sam Benkwitz-Bedford, Martin Palm, Talip Yasir Demirtas, Ville Mustonen, Anne Farewell, Jonas Warringer, Leopold Parts, Danesh Moradigaravand

**Affiliations:** a Center for Computational Biology, Institute of Cancer and Genomic Sciences, University of Birmingham, Birmingham, United Kingdom; b Department for Chemistry and Molecular Biology, University of Gothenburg, Gothenburg, Sweden; c Centre for Antibiotic Resistance Research at the University of Gothenburg, Gothenburg, Sweden; d Organismal and Evolutionary Biology Research Programme, Department of Computer Science, Institute of Biotechnology, University of Helsinki, Helsinki, Finland; e Helsinki Institute for Information Technology HIIT, Helsinki, Finland; f Wellcome Sanger Institute, Wellcome Genome Campus, Hinxton, Cambridgeshire, United Kingdom; g Department of Computer Science, University of Tartu, Tartu, Estonia; California State University, Fresno

**Keywords:** antimicrobial resistance, deep learning, high-throughput assay, machine learning, whole-genome sequencing

## Abstract

Escherichia coli is an important cause of bacterial infections worldwide, with multidrug-resistant strains incurring substantial costs on human lives. Besides therapeutic concentrations of antimicrobials in health care settings, the presence of subinhibitory antimicrobial residues in the environment and in clinics selects for antimicrobial resistance (AMR), but the underlying genetic repertoire is less well understood. Here, we used machine learning to predict the population doubling time and cell growth yield of 1,407 genetically diverse E. coli strains expanding under exposure to three subinhibitory concentrations of six classes of antimicrobials from single-nucleotide genetic variants, accessory gene variation, and the presence of known AMR genes. We predicted cell growth yields in the held-out test data with an average correlation (Spearman’s ρ) of 0.63 (0.36 to 0.81 across concentrations) and cell doubling times with an average correlation of 0.59 (0.32 to 0.92 across concentrations), with moderate increases in sample size unlikely to improve predictions further. This finding points to the remaining missing heritability of growth under antimicrobial exposure being explained by effects that are too rare or weak to be captured unless sample size is dramatically increased, or by effects other than those conferred by the presence of individual single-nucleotide polymorphisms (SNPs) and genes. Predictions based on whole-genome information were generally superior to those based only on known AMR genes and were accurate for AMR resistance at therapeutic concentrations. We pinpointed genes and SNPs determining the predicted growth and thereby recapitulated many known AMR determinants. Finally, we estimated the effect sizes of resistance genes across the entire collection of strains, disclosing the growth effects for known resistance genes in each individual strain. Our results underscore the potential of predictive modeling of growth patterns from genomic data under subinhibitory concentrations of antimicrobials, although the remaining missing heritability poses a challenge for achieving the accuracy and precision required for clinical use.

**IMPORTANCE** Predicting bacterial growth from genome sequences is important for a rapid characterization of strains in clinical diagnostics and to disclose candidate novel targets for anti-infective drugs. Previous studies have dissected the relationship between bacterial growth and genotype in mutant libraries for laboratory strains, yet no study so far has examined the predictive power of genome sequence in natural strains. In this study, we used a high-throughput phenotypic assay to measure the growth of a systematic collection of natural Escherichia coli strains and then employed machine learning models to predict bacterial growth from genomic data under nontherapeutic subinhibitory concentrations of antimicrobials that are common in nonclinical settings. We found a moderate to strong correlation between predicted and actual values for the different collected data sets. Moreover, we observed that the known resistance genes are still effective at sublethal concentrations, pointing to clinical implications of these concentrations.

## INTRODUCTION

Escherichia coli is a dominant bacterial species in the lower intestine of humans and other endotherms, as well as within a range of environmental niches (1). Over recent decades, the rising frequencies of E. coli strains that are resistant to multiple antimicrobials in both the clinic and the environment have become a source of serious concern for human and livestock health (2). A remarkably broad and flexible genetic repertoire spanning both the core and the accessory genome appears to underlie the rapid spread of multidrug-resistant E. coli. To diagnose and understand this spread, we must accurately and exhaustively estimate how the variants in this genetic repertoire, individually and in combination, affect E. coli growth under exposure to the range of antimicrobial concentrations that the species encounters in nature and in the clinic.

The use of the E. coli gene knockout (KO) library has captured the effect of the complete loss of many individual genes in the K-12 genome on growth in sublethal antimicrobial concentrations (3, 4). Combined with mechanistic modeling, such data can guide our understanding of some of the K-12 antimicrobial resistance (AMR) defense systems. However, natural E. coli strains have an open pangenome, which results in a very high level of population diversity (5). The genome of K-12 may not represent this diversity well, given that the phylogroup of K-12 only harbors ∼20% of genes across the pangenome for known E. coli strains and a similar fraction of the single-nucleotide diversity present in the species (6, 7). As a result, most AMR effects likely evade detection in K-12 screens, while those that are detected often have little or no effect in other backgrounds. Genome-wide association studies on broader panels of clinical and environmental E. coli strains could successfully estimate moderate or large AMR effects of common variants of all types (8). However, rare, weak, and background-dependent AMR effects are challenging to detect and measure. Linkage also means that some detected variants, although they may serve as diagnostic biomarkers for AMR, provide little molecular understanding of AMR itself. Moreover, these methods examine the association of the presence/absence of variants with the phenotype in isolation from the rest of the variants, thus neglecting potential effects of interactions between variants (9).

Machine learning methods incorporate genomic variants into a single prediction framework that can capture the background dependency of AMR effects. In recent years, the number of machine learning models for predicting AMR from whole-genome sequencing data has risen sharply (10–13). Ensemble models that combine results from multiple weak learners into one model proved superior to other models (14). So far, most studies have focused on qualitative AMR data sets with strains classified as resistant or susceptible to diagnostic antimicrobial concentrations or MICs, and models are judged based on how accurately this binary classification is recalled. The limited scope means that much of the underlying biological variation has been obscured (reviewed in Liu et al. [12]). As a result, published approaches have mostly captured common and large AMR effects, which represent already well-understood aspects of AMR biology, while the impact of rare, weaker, or background-dependent contributions on bacterial growth under AMR treatment has generally been neglected (12). Furthermore, the evolution of antimicrobial resistance in nature is likely driven by antimicrobial concentrations that are much lower (<1%) than the MIC or the diagnostic concentrations used in the clinic (15). Antimicrobials can reach high local concentrations downstream of production plants and sewer outlets (16), but are diluted to a 100-fold below the MIC in many environmental and wild animal niches where E. coli is more frequent (15–17). It is therefore entirely conceivable that the natural selection for AMR resistance at subinhibitory concentrations may be driven partially, or even largely, by effects other than those controlling resistance at diagnostic concentrations.

Here, we employed machine learning models to quantitatively predict the population doubling time and cell growth yield of >1,400 E. coli strains at subinhibitory antimicrobial concentrations from genomic data with no prior information on the resistance mechanism. We quantified the contribution of both known AMR determinants and genetic variants previously not known to affect AMR, disclosing the general importance of cell wall biosynthesis. Despite the unprecedented scale of the study and the low measurement error, the best model predictions were limited to an average correlation (Spearman’s ρ) of 0.63 (ranging from 0.36 to 0.81 across antimicrobial treatment conditions) for growth yield and 0.59 (ranging from 0.32 to 0.92 across antimicrobial treatment conditions) for doubling time across antimicrobials. The unaccounted heritability of antimicrobials resistance is therefore substantial, underscoring the challenge of fully explaining AMR in an enormously diverse bacterial species in which most causal variants are rare or only rarely affect AMR.

## RESULTS

To predict E. coli growth at subinhibitory concentrations of antimicrobials, we used >1,400 clinical, commensal, and environmental E. coli strains from major globally circulating clones in the TransPred project (www.github.com/matdechiara/TransPred). We used available whole-genome sequence information and data on the population doubling time and cell growth yield of each isolate when clonally expanding as observations in our prediction framework. We selected three subinhibitory concentrations of six bacteriostatic and bactericidal antimicrobials effective against E. coli as measurement contexts.

**FIG 1:**
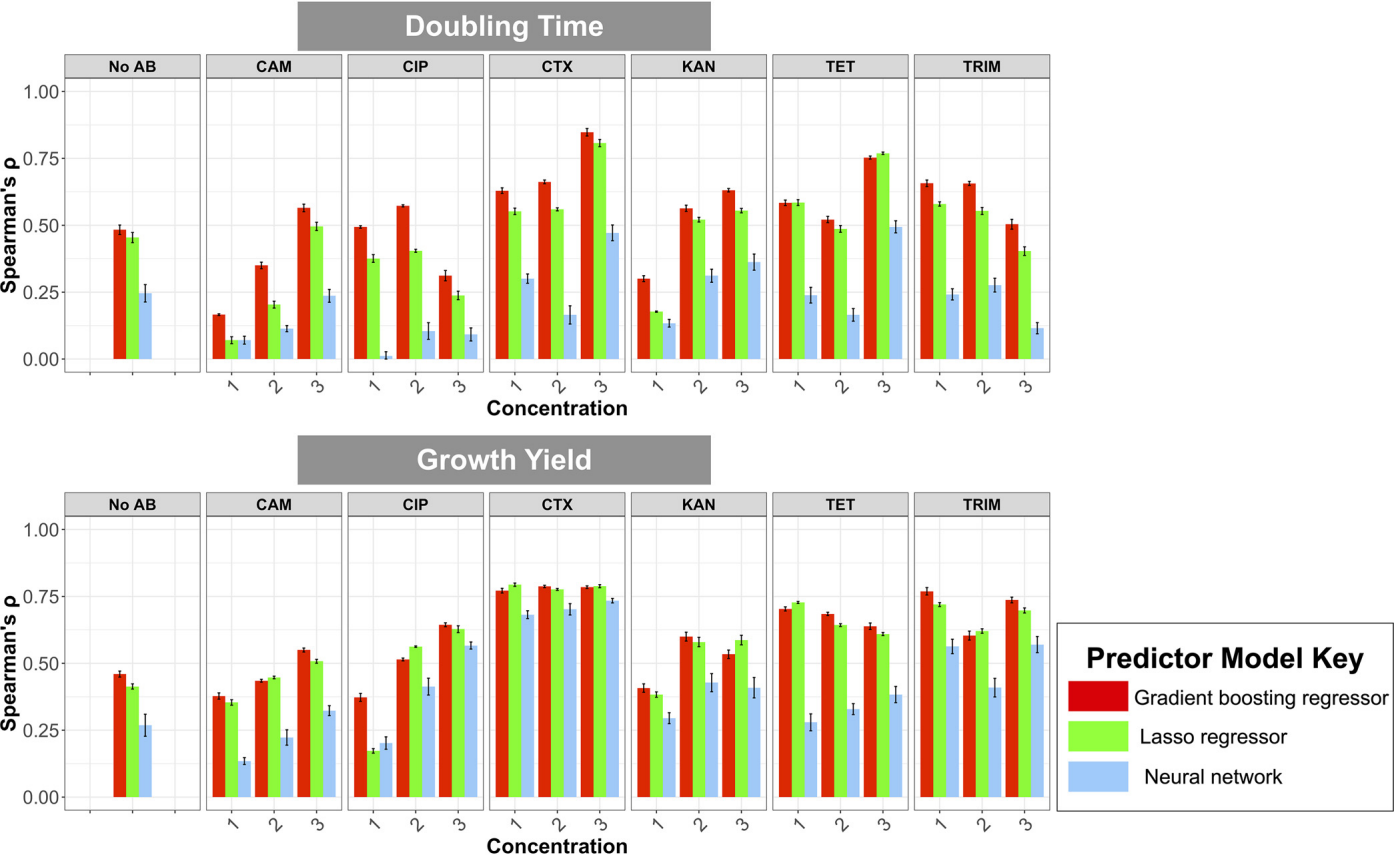
The performance (Spearman’s ρ, *y* axis) of the best-performing predictive models of each model type (colors) for 6 antimicrobials (panels, *x* axis) at 3 concentrations (*x* axis) and under the control condition of no antimicrobial treatment (no AB) for doubling time (top row) and growth yield (bottom row). The performance was assessed as the magnitude of correlation (Spearman’s ρ) between the predicted and real data in the test data set. Numbers 1, 2, and 3 represent low, medium, and high subinhibitory concentrations of antimicrobials, respectively. Values are corrected for the measurement errors. Error bars for the gradient boosted and lasso regressors correspond to 95% confidence interval computed from Spearman’s ρ values for four cross-validation data sets. Error bars for the neural network shows 95% confidence interval computed from Spearman’s ρ values for 10 independent runs of the best-performing models on the test data sets.

In E. coli, resistance to diagnostic concentrations of the antimicrobials used here occurs mainly through the horizontal transfer of plasmid-borne accessory genes that vary in presence/absence across strains. We therefore first probed how well we could predict growth at subinhibitory antimicrobial concentrations from the pangenome with linear, gradient-boosting, and neural network regressors. The pangenome was composed of 47,717 genomic features with unique presence-absence distributions of gene families across our strains. Based on gene presence-absence data, our best-performing predictive models for each class of models attained means of 0.53 (range, 0.13 to 0.79) and 0.40 (range, 0.01 to 0.85) of Spearman’s ρ for growth yield and doubling time, respectively, in held-out test data (Fig. 1). The correlations between predicted and actual values from the best-performing model were all significant across antimicrobials and conditions compared to the median correlation value computed for randomized data sets (see Fig. S1 in the supplemental material). The observed improvements in Spearman’s ρ values when we compared predicted value with randomized values were, on average, 0.35 (range, 0.07 to 0.65) for population doubling time and 0.54 (range, 0.12 to 0.71) for growth yield (Fig. S1). In 6 out of 12 conditions with antimicrobial treatment for growth yield and doubling time, respectively, the measures monotonically improved with increasing antimicrobial concentrations. This pattern is likely due to the higher fraction of between-strain variation explained by genetics, i.e., a higher broad-sense heritability, at these concentrations (Fig. 1). Among predictive models, in 6 out of 38 conditions, the lasso regressors were superior (Fig. 1). Neural networks consistently performed more poorly than other models, suggesting that with the current sample size, the incorporation of complex feature interactions did not improve predictions. The gradient-boosting regressor outperformed lasso and neural networks for 32 out of 38 conditions (Fig. 1), underscoring the suitability of these models for genome-based prediction of AMR, as reported previously (11, 18, 19). Henceforth, we employed the gradient boosting regressors for the downstream analyses.

10.1128/mSystems.00346-21.1 FIG S1 The significance of prediction results for the best-performing tuned gradient regressor model shown in Fig. 1. The boxplots show the distribution of coefficient of correlation between 100 bootstrapped label datasets from the actual data and the predicted labels from the models. The red lines show the values for the original prediction performance for the model. Download FIG S1, PDF file, 0.2 MB. Copyright © 2021 Benkwitz-Bedford et al. 2021 Benkwitz-Bedford et al. https://creativecommons.org/licenses/by/4.0/ This content is distributed under the terms of the Creative Commons Attribution 4.0 International license.

Prediction error rates for the training data sets were lower than those for the test data sets, (Fig. S2), and many plausible causes could be responsible, including that the number of predictors features vastly exceeds the sample size. Feature importance analysis revealed that only <0.02% of genome features were consistently picked by the models across cross-validation data sets, ruling out feature selection as an effective means for reducing the number of predictors (Fig. S3A). Moreover, using dimension reduction for reducing the number of predictors led to higher overfitting and lower accuracy (Fig. S3B). We therefore conclude that many predictor features, each of low importance, contribute to prediction accuracy and therefore that all information, without reduction, should be used for training models for best performance.

10.1128/mSystems.00346-21.2 FIG S2 The mean absolute error (MAE) values and correlation coefficient for training and test datasets for the best-performing gradient regressor models shown in Fig. 1. Error bars show standard deviation computed from four training and cross-validation datasets. Download FIG S2, PDF file, 0.3 MB. Copyright © 2021 Benkwitz-Bedford et al. 2021 Benkwitz-Bedford et al. https://creativecommons.org/licenses/by/4.0/ This content is distributed under the terms of the Creative Commons Attribution 4.0 International license.

10.1128/mSystems.00346-21.3 FIG S3 The sharing of important features across models trained for four cross-validation training datasets and principal-component (PC) based prediction. (A) Bars show the relative frequency of occurrence of important features across four cross-validation datasets under different conditions for antimicrobial treatments and measured features (growth yield and doubling time). (B) PC-based prediction of ceftriaxone (CTX) growth yield under high antimicrobial concentrations compared with the prediction based on the whole dataset. Bar plots show the error (MAE) and correlation coefficient (Spearman’s ρ) values for gradient-boosted regressors with different hyperparameter values in the grid search space. The maximum number of PCs was 845. Download FIG S3, PDF file, 0.3 MB. Copyright © 2021 Benkwitz-Bedford et al. 2021 Benkwitz-Bedford et al. https://creativecommons.org/licenses/by/4.0/ This content is distributed under the terms of the Creative Commons Attribution 4.0 International license.

Despite a large data set, even the best-performing model failed to explain, on average, 44% of the variance across conditions when only taking accessory gene variation into account. This performance was obtained after accounting for measurement variance. The average Spearman’s ρ between bootstrapped replicates was 0.76 (standard deviation, 0.14; see Materials and Methods) across antimicrobial concentrations and measurements, showing low levels of environmental variation and stochastic noise. Thus, most of the unexplained variation is genetic in nature and reflects a missing heritability. Conceivably, accessory genes that are too rare, too weak, or too dependent on the presence of other variants that are too rare to be captured by our models could account for some of this missing heritability. Such genetic effects stand a larger chance of being captured if the sample size is increased. We therefore examined whether moderate variations in sample size substantially impact prediction accuracy by downsampling the strains included in the predictor data set. We trained the models on randomly generated subsamples of our data set at different sizes and examined the performance of the trained on the same held-out test data set. Figure S4 reveals that for the majority of conditions the performance of the models tends to level out with increasing training sample size. We only detected monotonic improvement with increasing sample size for 6/19 (doubling time) and 6/19 (growth yield) conditions (Fig. S4A and S4B). These findings show that predictions are unlikely to improve substantially with moderate increases in sample size. Furthermore, the missing heritability is accounted for either by accessory genes whose effects can only be captured by vast increases in sample size or by genetic effects other than those associated with accessory genes (see Discussion).

10.1128/mSystems.00346-21.4 FIG S4 The mean absolute error (MAE) values for training and test data for the tuned gradient regressor model. Each box shows the results for 100 randomly generated subsamples at the sizes indicated on the *x* axis. We used the gradient-boosted regressor model with 100 iterations and default parameters values for tree depth and subsamples. Download FIG S4, PDF file, 1.7 MB. Copyright © 2021 Benkwitz-Bedford et al. 2021 Benkwitz-Bedford et al. https://creativecommons.org/licenses/by/4.0/ This content is distributed under the terms of the Creative Commons Attribution 4.0 International license.

**FIG 2:**
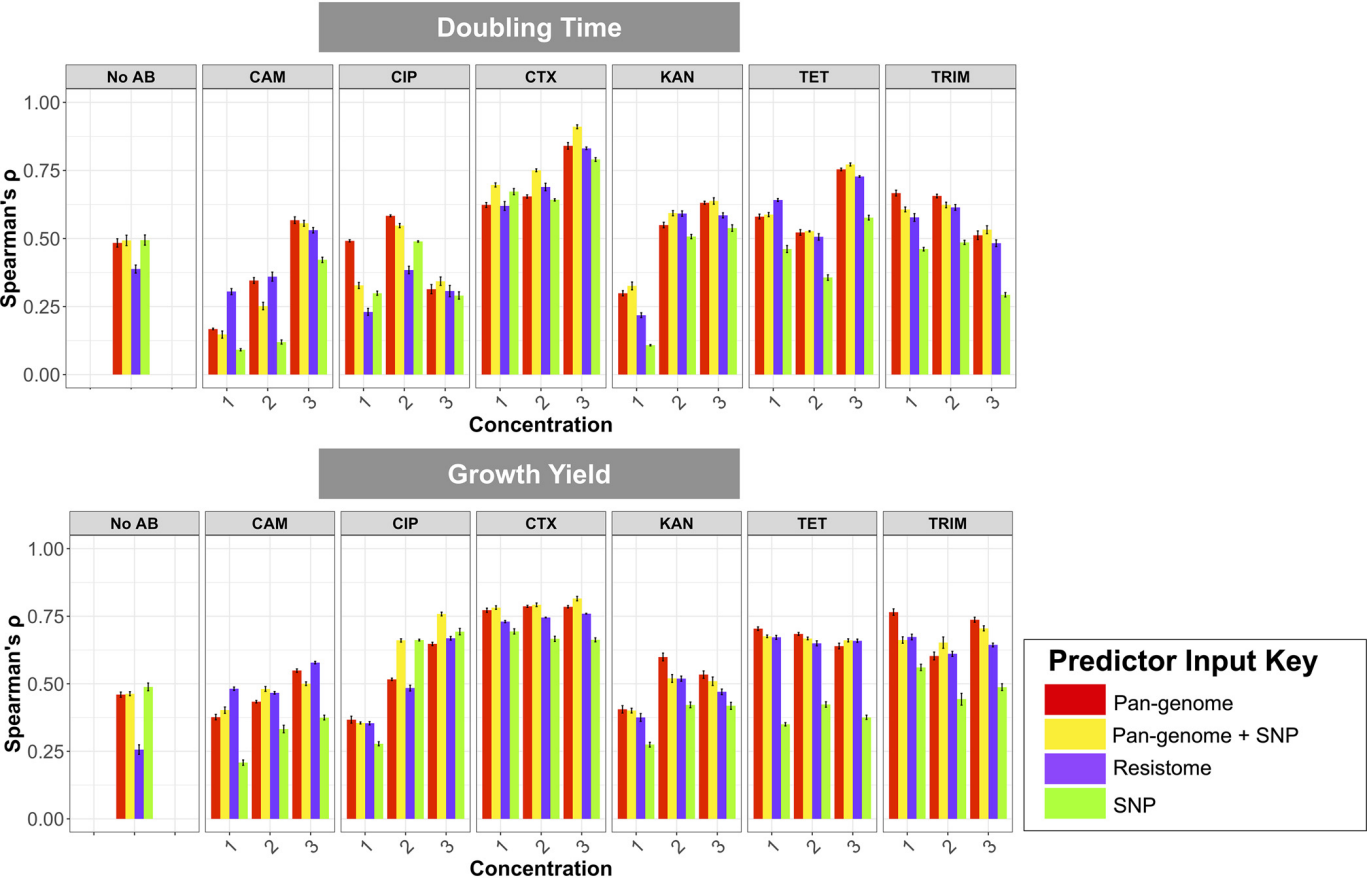
The performance (Spearman’s ρ, *y* axis) of the best-performing gradient-boosted regressor model of each predictor feature sets (colors) for 6 antimicrobials (panels, *x* axis) at 3 concentrations (*x* axis) and under the control condition of no antimicrobial treatment (no AB) for doubling time (top row) and growth yield (bottom row). The performance was assessed as the magnitude of correlation (Spearman’s ρ) between the predicted and real data in the test data set. The values are corrected for the measurement errors. Numbers 1, 2, and 3 represent low, medium, and high subinhibitory concentrations of antimicrobials, respectively. Error bars correspond to 95% confidence interval computed from Spearman’s ρ values for four cross-validation data sets.

We pursued the latter hypothesis by asking to what extent predictions of growth at subinhibitory antimicrobial concentrations improved by including the presence and absence of individual SNPs in the core and noncore E. coli genomes. We included 879,037 SNP features in training data set for training gradient-boosting models (Fig. 2). The inclusion of SNP information improved the pangenome-based prediction for 12/19 and 9/19 conditions for population doubling time and growth yield, respectively. The average prediction improvement over the pangenome-based prediction was 5.5% (range, 0.8% to 12%) for population doubling time and 6.9% (range, 0.7% to 21%) for growth yield. Because the SNP input matrix retains information on the presence and absence of genes, as we have examined SNPs in the accessory genome as well, the prediction accuracy of models based on SNPs alone could not be strictly evaluated. We conclude that SNPs whose effects are sufficiently common and strong to be captured by modeling on sample sizes of >1,400 genotypes only accounts for a small fraction of the missing heritability of AMR in our data set.

The current standards for predicting antimicrobial resistance at diagnostic concentrations rely on detecting the presence of known resistance genes, together called the resistome (20). We therefore evaluated how well models based exclusively on the known resistome performed compared to models that used more complete genome information. We found that predictions of models using only the resistome data better predicted the cell growth yield than the population doubling time under 13/18 antimicrobial treatment conditions, suggesting that antimicrobial resistance genes generally enhance the cell growth yield more than they reduce the population doubling time (Fig. 2). This is attributable to the fact that growth yield values have a broader range than that of doubling time (Fig. S4A), resulting in a stronger signal-to-noise ratio and consequently a better performance of predictive models. The prediction performance worsened for 16/19 growth yield and 16/19 doubling time conditions when we used the resistome alone; that is, the models achieved a worse prediction performance than what the best-performing models achieved using the complete genome information, i.e., considering all SNPs and gene presence-absence variations (Fig. 2). We conclude that E. coli growth at subinhibitory antimicrobial concentrations is generally predicted best from whole-genome data. Furthermore, our results overall indicate that, across different data sets, gradient-boosting regressors yielded Spearman’s ρ values with an average of 0.59 (range, 0.30 to 0.92) for population doubling time and 0.63 (range, 0.36 to 0.81) for growth yields (Fig. 2).

AMR in clinical contexts is diagnosed as growth or lack of growth at high MICs. To explore to what extent antimicrobial resistance at subinhibitory concentrations and MIC is predictable from the same genomic features, we labeled our strains as “resistant” or “susceptible,” based on the distributions of predicted doubling times and growth yields (Fig. S5A and B) (see Materials and Methods). We next measured how well these labels recalled and predicted those assigned for the same strains grown at higher MICs. Our results showed that resistance sensitivity labels assigned based on growth yield at subinhibitory concentrations captured those assigned at high MICs with accuracies that differ substantially between antibiotics. The average recall rates were 0.78 (for ceftriaxone [CTX]), 0.84 (for ciprofloxacin [CIP]), and 0.73 (for trimethoprim [TRIM]), and the average precisions were 0.87 (for CTX), 0.35 (for CIP), and 0.89 (for TRIM) (Fig. S5C). Labels assigned based on population doubling times at MICs captured labels at higher concentrations less well, with average recall rates of 0.72 (for CTX), 0.28 (for CIP), and 0.08 (for TRIM) and average precisions of 0.74 (for CTX), 0.21 (for CIP), and 0.27 (for TRIM) (Fig. S5C). Resistance to diagnostic concentrations of CTX and TRIM, which is known to be determined mainly by the presence of certain accessory resistance genes, was better predicted by inferred growth measures at subinhibitory than resistance to CIP, which is held to be primarily driven by chromosomal mutations in core E. coli genes (Fig. S5C). We compared the performances of best-performing prediction models for growth yield at subinhibitory concentrations with those of previous machine learning models that were trained on large-scale pangenomes and binary phenotypic labels (resistant versus susceptible) (18). Subinhibitory concentration-based models for growth yield values attained 89% of reported precision and 76% of reported recall for TRIM and 95% of reported precision and 99% of reported recall for CTX, compared to those of the best-performing predictive models in Moradigaravand (18). This shows that for particular groups of antimicrobials, E. coli growth yields predicted at subinhibitory antimicrobial concentrations can capture AMR phenotypes at diagnostic concentrations very well.

10.1128/mSystems.00346-21.5 FIG S5 (A) Distribution of fitness assay data for doubling time and growth yield. (B) Example of the predicted values for the growth yield for CTX under high antimicrobial concentrations. The dotted line shows the fitted curve for a bimodal distribution. The green and red lines show the inferred underlying normal distributions, used to assign resistant and susceptible labels, respectively. (C) Prediction of the resistant phenotypes using labels inferred from the predicted bimodal distributions for three antimicrobials and three concentrations. Download FIG S5, PDF file, 0.4 MB. Copyright © 2021 Benkwitz-Bedford et al. 2021 Benkwitz-Bedford et al. https://creativecommons.org/licenses/by/4.0/ This content is distributed under the terms of the Creative Commons Attribution 4.0 International license.

**FIG 3:**
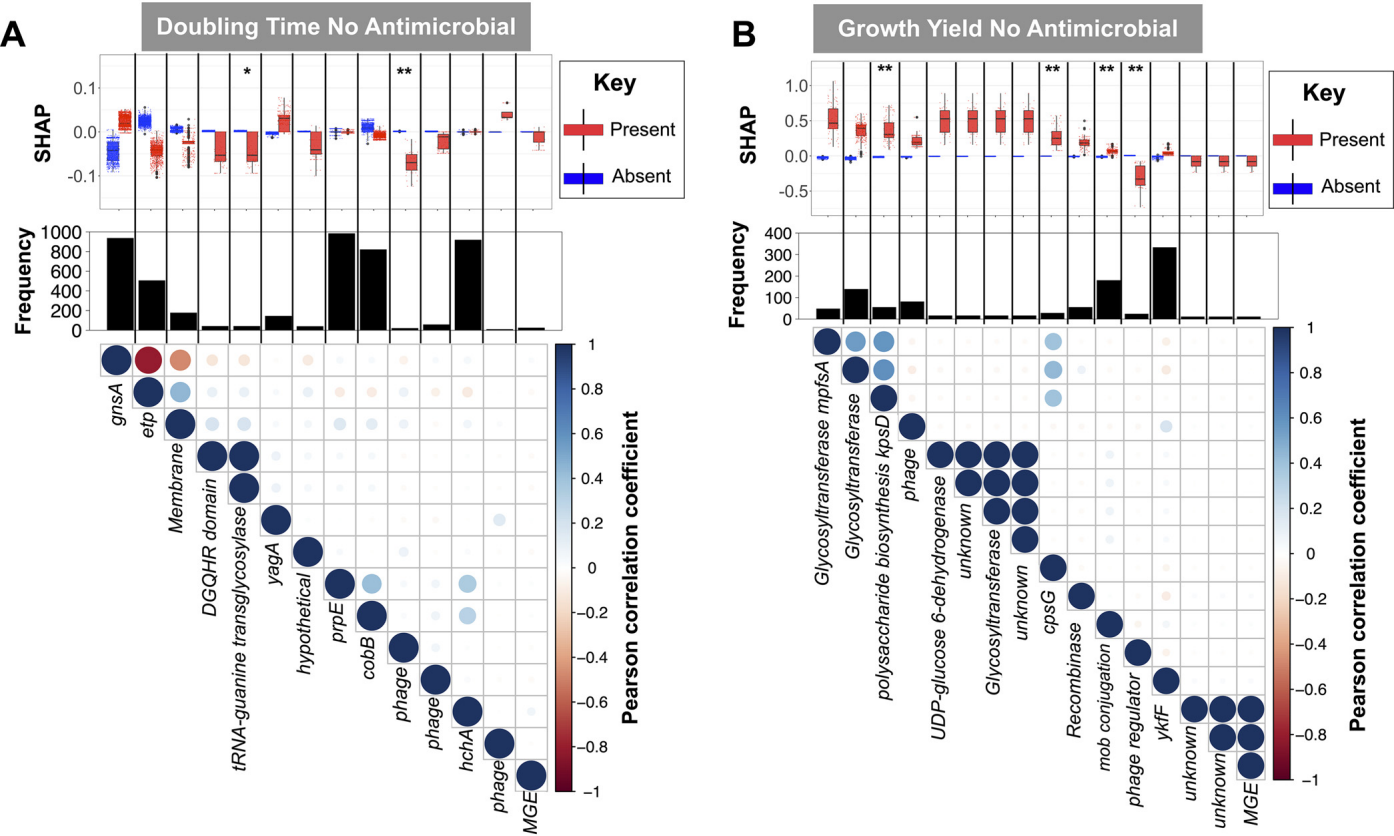
Feature importance in gradient-boosted regressor models for (A) growth yield and (B) doubling time in the absence of antimicrobials. Features are predictive gene family features, which are sorted according to their average ranks across models trained on four cross-validation subsets. Box plots show the Shapley additive explanations (SHAP) value, i.e., the effect of the presence or absence of the genes on the response features of doubling time and growth yield. Bar plots show the frequency of the gene families in the pangenome. Asterisks indicate the significance of *P* values for independence from population structure, computed by Scoary, at 0.05 (*) and 0.01 (**) levels. The matrix shows pairwise association between the presence of hits, where the color density shows the strength of association and colors show the direction of the Pearson correlation. The sequences of the gene families are provided in GitHub directory of the project.

We next sought to understand which gene presence and absence variation contributed to predicting E. coli growth (see Materials and Methods). We first identified genes important to population doubling time and growth yield in the absence of antimicrobials. We found 16 (growth yield) and 14 (population doubling time) genomic features (individual genes and sets of gene presence-absence variations) that the gradient-boosting models consistently used for prediction (Fig. 3A and B). Shapley additive explanation (SHAP) plots confirmed that the presence of 12/16 (growth yield) and 3/14 (doubling time) of these features was associated with better growth (Fig. 3A and B). None of these genomic features were shared between growth yield and doubling time, consistent with these being genetically distinct aspects of E. coli growth. Most of the detected genes lacked an annotated function or were annotated as mobile genetic elements and/or phage genes. A few features (2/14 for cell doubling time and 7/16 for growth yield) cooccurred across strains, i.e., Pearson’s *r *> 0.90 for their associations with each other), excluding an extensive effect of the linkage between genetic features (Fig. 3A and B). The majority of identified genes, i.e., 12/16 and 12/14 of features for growth yield and doubling time, respectively, were also distributed across clades in a manner that was associated with the population structure (*P* value from association, >0.05). Among features not linked with population structure for doubling time, we identified only a copy of the metabolism gene encoding tRNA-guanine transglycosylase (Fig. 3A). For growth yield, we identified three copies of the glycotransferase and polysaccharide translocation channel gene (*kpsD*) (21), all of which are implicated in cell wall biosynthesis, to be significantly independent from population structure (Fig. 3B).

**FIG 4:**
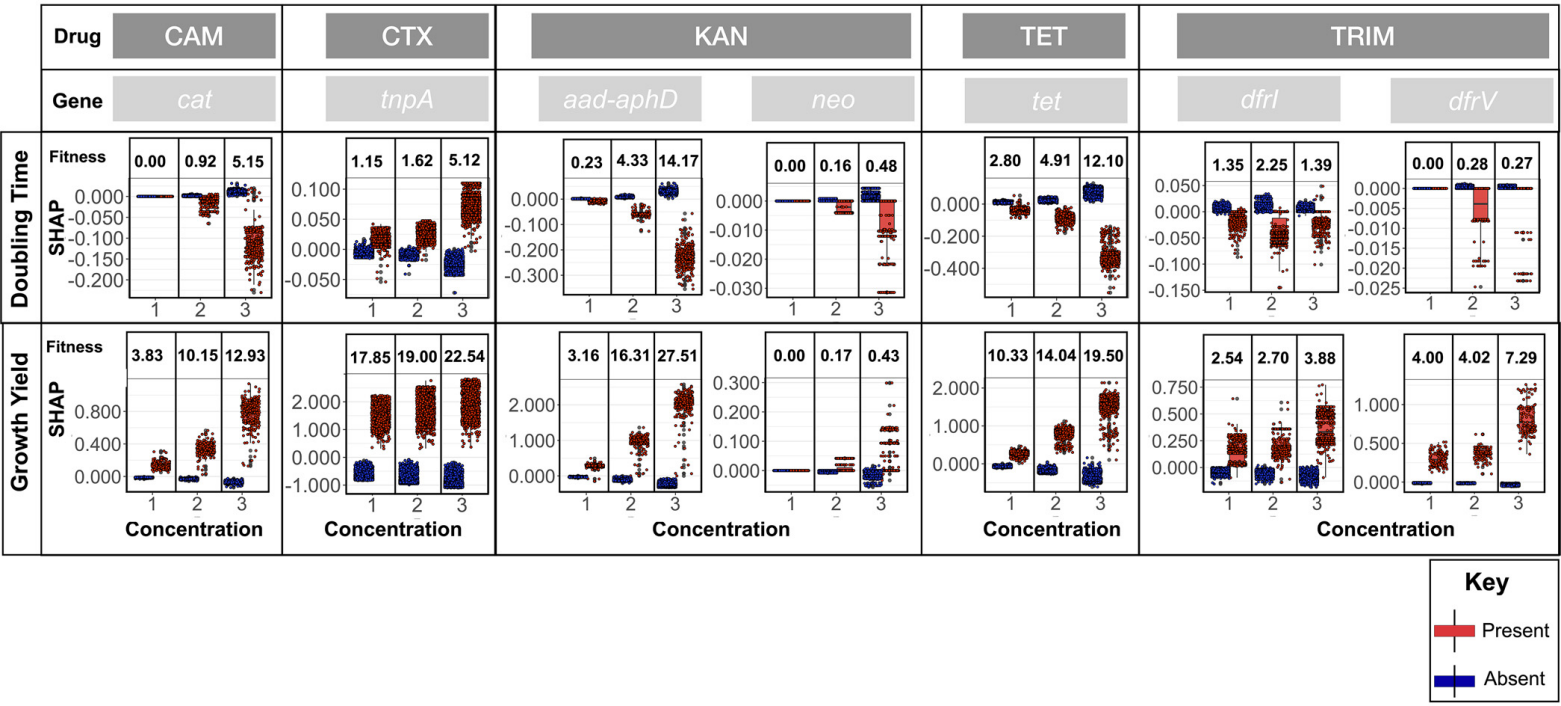
The contribution of the presence of known resistance genes and *tnpA* gene to the prediction of growth yield and doubling time, as measured by SHAP values. The *tnpA* gene was found to be linked with the extended-spectrum beta lactamase (ESBL) *bla*_CTX-M_ gene. Box plots in red and blue correspond to distribution of the effect of the presence and absence of features on growth-related features in each sample, respectively. The numbers above the box plot pairs show the difference between the medians of boxplots for the presence and absence under each condition divided by the range of values for the condition, which were turned into percentages. The numbers were used as proxies for the fitness effects of the resistance genes and the associated resistance gene for *tnpA*.

We next extracted the gene presence and absence features that predicted growth at subinhibitory antimicrobial concentrations and found between 141 and 99 accessory genes whose presences were always consistently utilized by models to predict growth across 18 antimicrobial treatment conditions for growth yield and doubling time, respectively. The distribution of most of these genes (114/141 for growth yield and 89/99 for doubling time, with a *P* value of <0.01 from Scoary) were at least somewhat linked with the population structure, making the latter a possible cause for their association to AMR. Among the genes with no significant lineage association, we found many known AMR gene families (Table 1 and Table S2). The presence of *aacA-aphD* and *neo* (kanamycin [KAN]), *cat* (chloramphenicol [CAM]), *tet* (tetracycline [TET]), and *dfrI* and *dfrV* (TRIM) resistance genes consistently predicted a higher growth yield in the presence of the expected drug, and the presence of *aacA* and *aphD* (KAN) and *tet* (TET) also consistently predicted a shorter population doubling time under the corresponding condition (Table 1 and Fig. 4). Our analysis also identified other gene families linked with resistance genes, e.g., the tetracycline repressor gene *tetR* for TET resistance and the alcohol dehydrogenase *frmA* gene for CTX resistance (Table 1). Being carried on plasmids that contain AMR genes also explains why genes required for plasmid transmission and replication and phage or transposons-linked genes predicted growth in some antimicrobials. Most notably, the *tnpA* encodes the transposase for transposon Tn*3* and has been reported in full or truncated forms downstream of diverse plasmid backgrounds containing *bla*_CTX-M_ gene family (22).

**TABLE 1:** Predictive biomarker gene families for doubling time and growth yield^*a*^

Gene	Product function	Linked ARGs^*d*^	Frequency	AMR^*c*^	Antimicrobial^*b*^																		
					None	CAM			CIP			CTX			KAN			TET			TRIM		
						1	2	3	1	2	3	1	2	3	1	2	3	1	2	3	1	2	3
*yigB*	Nuclease-like protein		75			G															G		
*vipB*	NAD-dependent epimerase/dehydratase, polysaccharide	*ugd*	65														G						
*tnpA*-*bla*	*bla*-linked MGE	*bla*_CTX-M_	382	CTX								G	G	G									
*tetR*	Tetracycline repressor protein	*tetA*	297															G	G	GD		G	G
*tetA*	Tetracycline efflux protein		304	TET														G	GD	GD	G	G	G
*sulI*	Dihydropteroate synthase type-1		377																	GD			
*rpoS*	RNA polymerase sigma factor		24														D					D	
*neo*	Aminoglycoside 3′-phosphotransferase	*aph*, *sul2*	268	KAN											G	G	GD				G		G
*mphR*	Regulator of macrolide 2′-phosphotransferase I		293										G										
*mphA*	Multidrug efflux transporter		295																		D		
*mhpF*	Acetaldehyde dehydrogenase		998															D					
*kpsD*	Polysaccharide biosynthesis/export protein		55		G																		
*folP2*	Dihydropteroate synthase	*aph*, *sul2*	197										G	G							G	G	
*fiu*	TonB-dependent receptor	*aad*, *sul1*	34									G											
*ebr*	Ethidium bromide resistance protein		375																		G	G	
*dhfrV*	Dihydrofolate reductase		51	TRIM																	G	G	G
*dhfrI*	Dihydrofolate reductase		263	TRIM																	G	G	G
*cpsG*	Phosphomannomutase (PMM)		28		G																		
*colE7*	Colicin activity protein		15						G	G													
*cmtR*	ArsR family transcriptional regulator		164																G				
*catI*	Chloramphenicol acetyltransferase		125	CAM		G	G	G															
*aacA*-*aphD*	Aminoglycoside adenylyltransferase		152	KAN											G	GD	GD						
*frmA*	Alcohol dehydrogenase	*bla*_TEM_	31																	G			
*glpR*	DeoR family transcriptional regulator	*bla*_SHV_	22									G	G	G									
*sat2*	TDP-fucosamine acetyltransferase	*aadA*, *dfrA*	77																				G
*cbiX*	Cobalamin biosynthesis protein		157										G										
*aph(6)-I*	Streptomycin phosphotransferase		151	KAN																D			
*ychA*	UPF0162 family protein		51										G										
UGDH gene	UDP-glucose 6-dehydrogenase	*ugd*	65														G						

a Found to be significantly linked with the phenotype after accounting for population structure for different treatment conditions, i.e., drug type and concentration (*P* value cutoff, <0.01). The full sequences of the genes are available in the GitHub directory of the project (see Materials and Methods). CAM, chloramphenicol; CIP, ciprofloxacin; CTX, ceftriaxone; KAN, kanamycin; TET, tetracycline; TRIM, trimethoprim.

b D, doubling time measurement; G, growth yield measurement.

c Antimicrobials for the known resistance genes. AMR, antimicrobial resistance.

d Known resistance genes in the Comprehensive Antibiotic Resistance Database (CARD) data set identified in the 200 bp downstream and upstream of the genes. The term ARG stands for AMR gene.

10.1128/mSystems.00346-21.9 TABLE S2 Definition of predictor accessory genes significantly unlinked with population structure. Download Table S2, CSV file, 0.02 MB. Copyright © 2021 Benkwitz-Bedford et al. 2021 Benkwitz-Bedford et al. https://creativecommons.org/licenses/by/4.0/ This content is distributed under the terms of the Creative Commons Attribution 4.0 International license.

The overlap between genomic features that were used to predict growth in different antimicrobial treatment conditions was low (average Jaccard distances of 0.02 and 0.04 for growth yield and cell doubling time gene features, respectively). Among the few genes whose presence predicted tolerance to multiple antimicrobials, we found well-known resistance genes, e.g., the amino 3′-glycosyl phosphotransferase (*neo*) gene and *tetA*, reflecting extensive coresistance, which in some cases is due to genetic linkage (Table 1). We used the SHAP values to quantify the impact of known resistance genes on the growth of each strain (Fig. 4). The presence of the tetracycline efflux pumps gene family *tet* improved the growth yield with 2.8%, 4.9%, and 12.1% of the total population range and reduced the cell doubling time with, on average, 10.3%, 14.0%, and 19.5% of the total population range for three increasing subinhibitory concentrations of the antimicrobial (Fig. 4). The impact of the presence of these genes is already noticeable at low tetracycline concentrations.

The two dihydrofolate reductase genes *dfrI* and *dfrV* both improved the growth yield under trimethoprim exposure, with average contributions of 3.04% and 5.10% of the total population range, respectively, but had only small effects on cell doubling time (Fig. 4). We examined the growth yield increases associated with *dfrI* and *dfrV* genes for different combinations of the presence and absence of the genes at three concentrations (Fig. S6). At all concentrations, the effect of either resistance gene on growth yield remained the same (for *dfrI*) or decreased slightly (for *dfrV*) in the presence of the other resistance gene (Fig. S6). The fitness interaction between resistance genes suggests a slight negative epistasis, consistent with the diminishing return of beneficial genes and variants reported in E. coli lab strains (21). The presence of the chloramphenicol acetyltransferase gene, *cat*, improved the growth yield by 3.8%, 10.1%, and 13.0% of the total population range at low, moderate, and high concentrations of chloramphenicol, respectively, but only marginally reduced the doubling time (5.5% at the highest concentrations) (Fig. 4). Similarly, the KAN resistance aminoglycoside phosphotransferase gene family *aacA-aphD* individually improved the growth yield by 3.6%, 16.3%, and 27.5% at low, moderate, and high kanamycin concentrations, respectively, but reduced the doubling time only slightly and only at the highest concentration (Fig. 4). Similarly to *aacA-aphD*, the presence of the *neo* gene increased the growth yield at intermediate and high concentrations, but at >1% of the effect of *aacA-aphD* (Fig. 4). The presence of the transposase *tnpA* gene, reflecting the impact of the linked with variants of *bla*_CTX-M_, was associated with an increased growth yield of 17 to 22% of the total population range across antimicrobial concentrations, but it had a consistently detrimental impact on the cell doubling time (Fig. 4). Overall, our predictions indicated that known resistance genes contribute mostly to growth yield rather than to doubling times. Moreover, the results provided quantitative measures of the growth effects of known AMR resistance genes for each strain.

10.1128/mSystems.00346-21.6 FIG S6 Fitness (growth yield) effects of trimethoprim (TRIM) resistance genes (*dhfrI* and *dhfrV*) for different genotypes of the resistance genes under three TRIM concentrations computed for models for four cross-validation training datasets. The 1 and 0 signs represent the presence and absence of the genes, respectively. Each box shows the distribution of Shapley additive explanation (SHAP) values for the genes for each genotype. Download FIG S6, PDF file, 0.1 MB. Copyright © 2021 Benkwitz-Bedford et al. 2021 Benkwitz-Bedford et al. https://creativecommons.org/licenses/by/4.0/ This content is distributed under the terms of the Creative Commons Attribution 4.0 International license.

While the contribution of gene presence-absence variation to AMR is relatively well understood, the contribution of SNPs is much less explored. Our analysis detected 67 synonymous, 17 nonsynonymous, and 9 intergenic significant variants (see Materials and Methods) on the predicted cell doubling time and growth yield. We detected known resistance determination mutations for CIP, including four variants, i.e., S83L, D87H, D87N, and D87Y, in the quinolone resistance-determining region of *gyrA* and one variant (S80I) in the *parC* gene (23). Besides CIP, the *gyrA* and *parC* mutations turned out to be somewhat predictive of the growth yield in subinhibitory concentrations of the other antimicrobials (Table S3). Given that our collection includes multidrug-resistant clinical strains, this observation indicates how the extensive coresistance for antimicrobials cause the presence of resistance variants for one antimicrobial to be predictive of resistance against other antimicrobials (see Discussion). Other nonsynonymous predictive mutations were found in genes encoding membrane and capsule proteins. These include an O-antigen of *wzxC* gene, a multitransmembrane protein with enormous sequence diversity that flips oligosaccharide substrates. Mutations that change O-antigen synthesis can have a significant effect on growth (24). The membrane protein genes with significant nonsynonymous mutations included the *nanC* (25) and *ytfQ* (26) genes, both of which are implicated in environmental nutrient uptake. The presence of membrane and capsule production genes in the SNP- and gene-based analysis suggests a key role for these genes in growth or stress response to antimicrobial treatment, although the causative link needs to be demonstrated in forward genetic experiments.

10.1128/mSystems.00346-21.10 TABLE S3 List of genes with nonsynonymous single-nucleotide polymorphism (SNP) biomarkers that were significantly linked (*P* < 0.05; analysis of variance [ANOVA]) with growth conditions measures under 38 conditions for doubling time and growth yield in the absence and presence (three concentrations) of six antimicrobials. Mutations in phage-related genes are not shown. The complete list of mutations is provided in the GitHub directory of the project. *G* and *D* correspond to growth yield and doubling time measurements, respectively. Download Table S3, PDF file, 0.1 MB. Copyright © 2021 Benkwitz-Bedford et al. 2021 Benkwitz-Bedford et al. https://creativecommons.org/licenses/by/4.0/ This content is distributed under the terms of the Creative Commons Attribution 4.0 International license.

Pangenomes show a wide range of sizes across species, with the accessory genome comprising 16% to 97% of the total for well-sampled genomes across bacterial species (27). We therefore examined whether variation in pangenome sizes would affect prediction, which informs about whether our models would be predictive in other bacterial species. In doing so, we repeated the prediction of growth yields at subinhibitory antimicrobial concentrations for simulated pangenomes with increasing accessory genome size and for increasing population size. As anticipated, the prediction error and the extent of overfitting monotonically decreased as population size increased (Fig. S7A), although a level of overfitting existed at all population sizes tested. The monotonic effect of decreasing pangenome size, and potentially an effect of the background noise from uncorrelated genes, on improving prediction could also be seen. When we decreased the pangenome size by 100 times, the true detection rate of the causative genes remained in the same range (49%, 46%, and 48% for gene acquisition values of 10^−7^, 10^−8^, and 10^−9^, respectively); however, an increase in sample size improved the detection rate (48%, 59%, and 61% for sample sizes of 200, 400, and 600, respectively) (Fig. S7B). Moreover, for a selective advantage as low as 0.5 of the causative gene, the correlation between predicted and actual values on the test data set was positive, irrespective of other pangenome parameter values. The value of 0.5 is greater than the estimated selective advantage for the resistance gene under 15 out of 19 treatment conditions for growth yields in Fig. S5A (see Materials and Methods). These results show that the predictive model remains applicable to a broad range of scenarios for pangenome evolution.

10.1128/mSystems.00346-21.7 FIG S7 Frequency of correct prediction of the causative gene for simulated pangenomes and increasing value for the selective advantage of 10 causative genes. White and black bars correspond to cases where the causative gene is correctly and incorrectly identified by the feature importance analysis of the tuned gradient boosted regressor model, respectively. Further details are provided in Materials and Methods. *N*, υ, and γ indicate population size, rate of gene acquisition, and rate of gene loss for reconstructed pangenomes, respectively. We used the gradient-boosted regressor model with 100 iterations and default parameters values for tree depth and subsamples. Download FIG S7, PDF file, 0.5 MB. Copyright © 2021 Benkwitz-Bedford et al. 2021 Benkwitz-Bedford et al. https://creativecommons.org/licenses/by/4.0/ This content is distributed under the terms of the Creative Commons Attribution 4.0 International license.

## DISCUSSION

In this study, we adopted a reverse genetics approach and applied three different machine learning models to predict bacterial growth and doubling time from genomic data under a range of growth conditions in natural E. coli strains. We focused on interpreting features of machine learning models to advance a mechanistic understanding of the genetic repertoire for growth under antimicrobial treatment.

Our results suggest different genetic repertoires for growth yield and doubling time. We note that growth yield in our study does not represent the total change in population size from start to end of growth, and henceforth it should not be seen as the efficiency with which the limited resource, i.e., carbon and energy, is utilized. Rather, the growth yield is an aggregate measure of growth, influenced by the dynamics of the growth rate along the entire growth curve, both in early and later phases of growth. Because an expanding cell population changes its own environment, by consuming nutrients and secreting by-products of metabolism, the genetic factors influencing growth have different weights on the growth rate at different time points. One example is nutrient uptake, in which low-affinity transporters are active at high nutrient concentrations and influence the growth rate at early stages of growth, while high-affinity transporters are active at low nutrient concentrations and influence the growth rate at later stages (28). Similarly, secretion of acidic metabolites generated by the tricarboxylic acid (TCA) cycle leads to an accelerating acidification of the surrounding environment as a colony expands (29). The local pH change affects the proton gradient across the membrane and thus the various processes that are driven by this gradient, including the activity of proton symporters and antiporters, e.g., major facilitator superfamily (MFS), multiantimicrobial extrusion protein (MATE), and resistance-nodulation-division (RND) transporters (30). At acidic pH, the proton gradient breaks down to the extent that drug export driven by proton influx becomes impossible, and the cell instead relies on ATPase efflux pumps and will consume energy in these processes. The growth at early and late stages, and consequently the cell doubling time and the growth yield, may therefore often be defined by different genetic factors.

We concluded that tree-based models were superior to standard fully connected neural network models for AMR prediction from genomic variants, confirming previous successful applications of ensemble-boosting classifier and regressor methods for inferring AMR phenotype and pathogenicity from genomic data in Gram-negative strains (11, 13). The superiority of ensemble-boosting methods to neural network models in genome-based AMR prediction tasks may be due to the nature of genomic data sets, in which predictive genomic features, particularly known AMR determinants, are individually meaningful and complex interactions between predictors are absent. For the purposes of routine and effective clinical use, these models still require interpretability, as the mechanisms and patterns that the model uncovers are important for practical applications. Our findings revealed how the interpretability of features could provide an understanding of the model when local explanations of each prediction were combined. Using this approach, we measured the marginal effect of biomarkers on prediction across different combinations of allelic status. Due to our modest sample size, we limited our analysis to known resistance determinants. However, the rapid increase in genomic data will allow future studies to extend the approach to infer fitness effects for many genomic variants with moderate to weak effects. Such analysis allows not only comparing the relative importance of resistance genes across conditions but assessing the average contribution of a gene across many genomic backgrounds, which is infeasible and laborious in the lab. The data-driven approach is also superior to experimentally assessing the fitness effects of genes in individual lab strains over the course of the evolution, since this approach accounts for various genetic backgrounds through which the gene is passed. By computing the average fitness effect and the extent of variation around that average, our approach becomes doubly valuable when considering the average effect in the presence or absence of other genes with an effect.

Antimicrobial resistance research has been largely focused on clinical strains and therapeutic antimicrobial concentrations. Therefore, the contribution of subinhibitory antimicrobial traces, released as a result of anthropogenic interventions in the environment, to the rise of resistance has not been sufficiently studied (31, 32). Prior evidence suggests that many types of plasmid resistance do not emerge *de novo* during treatment (33). Thus, a patient or animal is either infected with the susceptible bacterium, without any resistance developing during treatment, or they are infected with the resistant strain and the antimicrobial treatment mainly causes an enrichment of a preexisting resistance gene or mutation. Our results demonstrated that the impact of resistance genes is detected at subinhibitory concentrations. Therefore, the fitness effects appear to outweigh the fitness cost at low antimicrobial concentrations. This finding further supports the idea that the ability of most resistance genes to confer high-level resistance at a low fitness cost shields the selective dynamics of mutants at low drug concentrations, which leads to the selection and fixation of resistance variants (34). The result calls for the extension of the selection window in resistance stewardship programs to include subinhibitory antimicrobial concentrations, specifically the minimum selective concentration (MSC), in environmental sites (32, 35).

Despite attainment of strong average correlations of 0.63 and 0.59 between predictions for growth yield and population doubling time, respectively, there remains a large gap to perfect prediction. We do not attribute the low heritability to gene expression variation. The reason is that the variation, irrespective of the nature of the variation, i.e., environmental or stochastic, is expected to be captured in measurement errors, which we have accounted for in our models. Several other factors may explain the low heritability. Some studies have suggested a possible role for sequence-independent determinants of resistance, e.g., epigenetics, in the development of resistance to antimicrobials, particularly at subinhibitory concentrations (36–38). Another possible explanation is the role of rare variants or variants with weak fitness effects that remain to be discovered (39). These could be either common alleles with moderate effects or rare alleles with large effects. The latter has been reported for lab colony pools, where around 10% of resistance to rifampin was caused by numerous rare mutations (40). Furthermore, low-effect mutations differing from classic high-effect drug resistance have been identified to drive streptomycin resistance at subinhibitory levels in Salmonella enterica (41). Hence, capturing the information of rare genetic variants, including variant types caused by genomic rearrangements and insertion sequence movements (e.g., Di Gregorio et al. [42]), for the genome-based prediction of resistance remains a challenge (43). The total heritability may also be increased by genetic interactions (39) at inter- or intragene levels (44), which limits genome-based AMR prediction accuracy in small- to moderate-sized genomic data sets (43).

Besides the limitations of predictions, our results also showed the extent to which association of biomarkers with population structure and coresistance may constrain the application of ensemble models. The population structure association of predictive features causes the performance of the trained machine learning model to depend on the lineages included in the training data set. Comprehensive and large-scale genomic sampling is always required to mitigate the above effects. The extensive coresistance is caused by coselection of resistance determinants, which brings together resistance determinants for different drugs in the same genomic context. In natural settings, bacterial populations are exposed to a combination of antimicrobials, anti-infective agents, and heavy metals, all of which select for multidrug resistance (45). Coselective potential has been reported for a number of antimicrobials, e.g., CIP and TRIM, in microbial communities treated with subinhibitory concentrations of antimicrobials (46). For TRIM, coselection occurs as a consequence of sharing the same genomic context, i.e., class 1 integrons, of the *dhfr* gene with resistance genes for other antimicrobials. The presence of coselection between strains may improve the predictive model performance by providing further predictive signals in the data set that can be utilized by the model. However, coselection complicates the robust identification of a universal biomarker genes, as the models misidentify noncausative resistance genes as biomarkers. These challenges pose an issue for clinical diagnostic applications of genome-based machine learning frameworks.

Deriving a genotype-phenotype map for E. coli strains from diverse sources has important implications for understanding and predicting the dynamics of the population on epidemiological timescales and across environmental and clinical sites. Moreover, such a genotype-phenotype map may directly inform on potential novel targets. The successful application of machine learning methods provides the motivation for these methods to be employed in future studies to predict other clinically relevant traits, such as transmissibility, host preference, and horizontal gene transfer rate. These endeavors would meaningfully improve infectious disease diagnostics.

## MATERIALS AND METHODS

### Strains and genomic data.

We used data for 1,407 E. coli strains, which were collected, sequenced, and growth phenotyped in the ongoing TransPred project and that are available online as part of an expanding resource (www.github.com/matdechiara/TransPred). The strains were recovered from diverse human settings, including hospital- and community-onset infections, food, wild animals, and wastewater treatment plants. All TransPred sequencing was performed at the Wellcome Sanger Institute with a 450-bp insert size on Illumina HiSeq 2500 machine with paired-end reads with a length of 100 bp. For the purpose of predicting phenotypes from genomic information, we assembled the paired-end reads of these strains using an in-house Velvet-based (47) assembly and improvement pipeline (48). These *de novo* assemblies were annotated with Prokka (49). Codes and other intermediate files are available at www.github.com/dmoradigaravand/TransPred_ML.

### Genomic and phylogenetic analysis.

For the purpose of identifying genomic variants that are used by models for predictions of growth at subinhibitory antimicrobial concentrations, we mapped the short reads against the K-12 E. coli reference genome (accession number NZ_CP032667) with SMALT v0.7.4 (https://www.sanger.ac.uk/tool/smalt-0/). A threshold of 30 was used for mapping, and SNPs were subsequently called and annotated using SAMtools mpileup (50) and BCFtools (51). We removed SNPs at heterogeneous mapping sites in which the SNP was present in less than 75% of the reads at the site, as done previously (52).

To reconstruct the pangenome of the whole collection, we fed the output of Prokka into Roary (53) and used the default identity threshold of 95% to identify orthologous gene families. We utilized Scoary (54) with default parameters to compute the association between predictive accessory genes that were identified by the model and continuous growth features, i.e., population doubling time and cell growth yield, at each antimicrobial concentration. Since Scoary works on binary response features, we first binarized the continuous response variables according to the median value for each growth feature. We used the worst pairwise-computed *P* value in Scoary to reject the significance of phylogenetic signal, i.e., the association of the variant with the population structure (www.github.com/AdmiralenOla/Scoary). For the identified predictor genes that were found to be unlinked with the population structure, we further characterized resistance gene families. In doing so, we conducted a BLAST search with the default E value of 10 using the AMR genes in the Comprehensive Antibiotic Resistance Database (CARD) (55). To examine whether the predictor genes are tightly linked with any known AMR gene, we screened 200 bp downstream and upstream of the predictor genes for the presence of known AMR genes in CARD. We also explored the functions of top-hit genes that were identified by the predictive models by performing a BLASTX search against the NCBI protein database. AMR determinants were identified using ARIBA (56) with the default similarity threshold. The output of ARIBA was then turned into a presence/absence matrix.

### Growth at subinhibitory antimicrobial concentrations.

We used the population doubling time and growth yield data generated for the strains studied in the TransPred project (www.github.com/matdechiara/TransPred). The data capture the population doubling time and cell growth yield after 8 h of growth of 1,407 E. coli strains. Each colony grows on top of a solid matrix composed of LB medium, in which one of three sublethal concentrations for each of six antimicrobials, namely chloramphenicol (CAM; 0.5 μg/ml, 1 μg/ml, or 2 μg/ml), ciprofloxacin (CIP; 0.002 μg/ml, 0.004 μg/ml, or 0.008 μg/ml), kanamycin (KAN; 4 μg/ml, 8 μg/ml, or 16 μg/ml), tetracycline (TET; 0.3 μg/ml, 0.6 μg/ml, or 1.25 μg/ml), and trimethoprim (TRIM; 0.125 μg/ml, 0.25 μg/ml, or 0.5 μg/ml), had been embedded. Colonies were deposited as initially isogenic populations at initial population sizes of ∼100,000 cells, with 1,536 colonies deposited in systematic colony arrays on each plate, using robotics. A total of 384 of these colonies were identical controls used to correct for spatial bias between and within plates. For each concentration of each antimicrobial, each strain was cultivated as six biological replicates on different plates. Population expansion for each colony was followed by counting cells at 10-min intervals using the Scan-o-matic framework v2.0 (29). From each colony growth curve, the population doubling time and the total cell growth yield after 8 h were extracted. Experiments included automated transmission scanning and signal calibration in 10-min intervals, as described previously (29). The absolute population doubling times and growth yields were log_2_ transformed and normalized to the corresponding measures of adjacent controls on each plate, while data for missing or misquantified (growth curves heavily affected by technical artifacts, such as growth medium background features, evaporation, light source failures, or colonies expanding into each other, as reflected in abrupt peaks, collapses and other local irregularities in the measured light transmission) colonies were discarded. The median of these logged and normalized values across biological replicates for each lineage was retained and used as the continuous response variable in the machine learning setting. The accession number and the median and standard deviation of cell growth yield and population doubling time values for each strain is provided in Table S1 in the supplemental material.

10.1128/mSystems.00346-21.8 TABLE S1 Sample specifications and accession numbers. Download Table S1, CSV file, 1.3 MB. Copyright © 2021 Benkwitz-Bedford et al. 2021 Benkwitz-Bedford et al. https://creativecommons.org/licenses/by/4.0/ This content is distributed under the terms of the Creative Commons Attribution 4.0 International license.

The doubling time represents the minimal population doubling time achieved during the growth phase. The time that cell populations can sustain this rapid growth is typically short and occurs early, after 1.5 to 2 doublings. Before this phase, many cells remain in the lag phase that precedes reentry into the cell cycle, while after this phase, resources (in our case, carbon/energy) become insufficient to support maximal cell division for all cells in a population. The cell growth yield represents the total change in population size, from the start of the experiment to the 8-h time point at which we extract the yield. We note that the cell growth yield does not represent the total change in population size from start to end of growth, because cells often have not yet reached their maximal cell number at the 8-h time point.

### Machine learning to predict growth at subinhibitory antimicrobial concentrations based on the genome.

We calculated features from the genome to use as predictors in the models. These predictors included (i) pangenome gene presence, a predictor matrix that contains a binary indicator of gene presence in the strain from Roary for each accessory gene and for each E. coli strain; (ii) SNPs, a matrix that includes distinct encodings for alternative nucleotides and for the absence of the site compared with the reference genome; and (iii) the resistome (20), a predictor matrix that contains a binary indicator of the presence of an AMR determinant from CARD in the strain (55).

We applied three classes of machine learning methods on predictor matrices, a lasso-regularized regression model (57), a gradient-boosting regressor ensemble model (58), and a feed-forward residual neural network (59). The models were trained on 80% of the input data set, with 4-fold cross-validation for tuning hyperparameters, and evaluated on a 20% random held-out data set. For the lasso regression and gradient-boosted regressor models, we utilized the *Scikit-learn* package (60) and the functions “linear_model.Lasso” and “Ensemble.gradientBoostingRegressor,” respectively. We first measured the strength of the correlation between the predicted and actual data using the Spearman’s rank correlation coefficient (Spearman’s ρ) for four cross-validation data sets. We then conducted a one-sample Student’s *t* test on the distribution of coefficient values (Spearman’s ρ) for the validation data in four cross-validation data sets and retained models with a significantly positive coefficient value (*P* < 0.05). This step ensured that predicted values are positively linked with actual data, irrespective of the training data set. We then picked the hyperparameter set that yielded the minimum average value of the mean absolute error (MAE) estimates across four cross-validation data sets as the best-performing model.

The hyperparameter values for the best-performing model on the training/validation data set were then used to first train a model on the training data set and to examine its performance on the test data set. We reported the coefficient (Spearman’s ρ) of the correlation between the predicted values and actual value on the test data set as the performance of the model. We defined the strength of correlation based on Spearman’s ρ values as defined previously (61).

We used a grid search approach to find optimum values for the hyperparameters. The lasso model was tuned by finding the value for the penalty term by testing values (10^−5^, 10^−4^, 10^−3^, 10^−2^, and 10^−1^). We tuned the gradient-boosted regressors by finding the optimal values for key parameters, including tree depth (1–3), number of iterations (30, 60, or 100), and subsample size (1 or 0.8). The subsample parameter designates the fraction of samples used during the training of the model in each iteration. Subsample values of less than 1.0 result in stochastic gradient boosting. Manual inspection of error in runs over iterations revealed that models began to overfit after 100 generations for the response variable. We therefore limited the iteration numbers to values smaller than 100.

To develop the neural network, we used the Keras library (62) and trained fully connected feed-forward networks. We split the input data into test (20%), validation (20%), and training (60%) data sets, and tested *n *= 2, 4, or 6 hidden layers, and *n* = 20, 40, or 60 neurons per layer, which were screened jointly. We also examined the inclusion of a drop-out value of 0.2. The Adam algorithm was used for an adaptive learning rate optimization. Models were trained for at most 100 epochs, with a batch size of 32. We employed the “EarlyStopping” feature in the Keras library to terminate the training if the validation loss did not improve after 200 epochs. Moreover, we examined the inclusion of a “skip connection” feature in the model, which skips some layer in the neural network and feeds the output of the first layer as the input to the last layer. The skip connection has been suggested to mitigate the problem of vanishing gradients, and therefore, to speed up the learning process (63). Since the neural networks may yield different results across training runs, we repeated the training for each model specification 10 times and retained the models with a positive correlation between predicted and actual data in the training data set across all 10 runs. We then selected the model with the smallest value for the average MAE in the validation data set across 10 runs as the best-performing model.

Among different parameters for the neural networks, only the inclusion of the skip connection consistently improved the training accuracy, and other parameters appeared less effective. The inclusion of early stopping did not always improve the performance of models or the extent of overfitting, i.e., the difference between the error rate for training and test data set. The inclusion of the feature in the model was found to yield better validation MAE loss for ∼47% of the neural network models. Details of the performance of lasso, gradient-boosted regressor, and neural network models with hyperparameter values in the grid search space are provided in the GitHub directory (www.github.com/dmoradigaravand/TransPred_ML).

To account for the effect of measurement variations on prediction accuracy, we first computed the mean and standard deviation across six measurements for each strain under each condition. We then drew a sample from a normal distribution with the computed mean and standard deviation for the measurements for each sample. We repeated the process for all samples and created a sampled data set. We then measured Spearman’s ρ for correlations between the response feature values used for training the models and the sampled data. We repeated the process 100 times and calculated the mean of Spearman’s ρ values. We amended prediction performance values by dividing the correlation coefficient (Spearman’s ρ) from predictions by the calculated mean Spearman’s ρ for the sampled data.

We examined the effect of applying a dimension reduction preprocessing step to reduce the number of predictors. To this end, we first computed the principal components for the training data set and used these to transform the predictors in the test data set. We then used the new features for prediction. We conduced predictions with the gradient-boosting regressor models as detailed above. We screened a range of values for the number of PCs to examine the effect on both prediction accuracy and the extent of overfitting.

### Prediction of resistance and susceptibility labels at therapeutic concentrations.

We examined whether the population doubling time and growth yield predicted at subinhibitory antimicrobial concentrations reflected antimicrobial resistance at near-diagnostic MICs well. To do this, we used the phenotypic data reported in a previous study (18) on the resistance and susceptible labels, which was available for three drugs (CTX, CIP, and KAN), and a subset of 200 strains. In that study, we empirically measured whether each strain could grow at near-diagnostic MICs and labeled them accordingly as resistant and susceptible at therapeutic concentrations of antimicrobials. We then trained the gradient-boosted regressor models on a training/validation data set, excluding the 200 strains, and used the tuned model to predict cell yield and doubling time for the 200 strains with known phenotypic labels, i.e., resistant and susceptible. The distribution of growth-related features was bimodal, corresponding to resistant and susceptible strains, with modes separated more as the concentrations of antimicrobials increased (Fig. S5A). To convert continuous predicted values into binary values, we first fitted a bimodal distribution on predicted values with the expectation-maximization algorithm implemented in the “normalmixEM” function in the *mixtools* R library (64). We then used the posterior distributions for the fitted distributions to assign resistance and susceptible labels, assuming the distribution with a smaller mean corresponds to the susceptible subpopulation, as shown in an example in Fig. S5B. Since the algorithm may lead to different results, we repeated the fitting for 500 times and chose the fitted distributions in the majority of runs.

We then assigned labels as follows:

labelx=resistant if PxD1 > PxD2 susceptible otherwise

where *D*_1_ and *D*_2_ are parameters of the normal distributions of the two mixture components and $\mu_{D_{1}}\;>\;\mu_{D_{2}}$. We then measured precision (fraction of true-positive instances among the retrieved instances) and recall (fraction of retrieved true-positive instances among the true-positive instances) to assess the performance of classification.

### Extracting genomic features important to growth at subinhibitory antimicrobial concentrations.

We adopted global and local approaches to compute the importance of genomic features for predictions in the gradient-boosted regressor tree models. In the global approach, we used the feature importance calculator (feature_importances) as part of the *Scikit-learn* package, where genomic feature importance was computed during the optimization of the weak learner in the boosting process. Here, the importance value for a genomic feature corresponds to the fraction of samples for which the tree will traverse a node that splits based on the feature. These values were averaged across all of the trees during the iterations. In order to identify the features that most robustly contributed to prediction, we included only features that were selected by all models across four cross-validation training/validation data sets.

The local explanation approach employed the concept of Shapley additive explanations (SHAP), derived from coalitional game theory. The concept helps to explain the feature importance for the output for each sample. The SHAP method is an additive feature attribution method in which predictor feature values of data points serve as players in a coalition. A player is an individual accessory genome feature. The Shapley value of a feature value is defined as the contribution of the predictor to the payout, i.e., prediction, which is weighted and integrated over all possible feature value combinations. Shapley values are computed by introducing each feature, one at a time, into a conditional expectation function of the model’s output, $f_{x}\left(S\right)=E\langle f(X)|\mathrm{do}\left(X_{s}=x_{s}\right)\rangle$, and attributing the change produced at each step to the feature that was introduced, then averaging this process over all possible feature orderings. Here, *S* denotes the set of predictor features on which we are conditioning, *X* is a random variable representing the model’s input predictor features, and x is the model’s input vector for the prediction. We used the “TreeExplainer” function to explain the fitted tree as implemented previously (65), available in www.github.com/slundberg/shap.

Besides single predictors, the SHAP method allows to simultaneous determination of the growth effect for the combination of multiple resistance gene predictors. We obtained such combined effects of specific antimicrobial genes for TRIM, for which we identified two well-characterized resistance genes, *dfrI* and *dfrV*, on growth yield. In doing so, we measured SHAP values for single resistance genes in different contexts resulting from four combinations of the presence and absence of TRIM resistance genes, i.e., *dfrI* and *dfrV*.

To assess the prediction importance of SNPs, we first extracted the global importance values for SNP predictors consistently used by the gradient-boosting regressor model, as detailed above. Since we included SNPs in both the core and accessory genomes, we specifically tested whether the presence of a base pair substitution at the site, and not the presence/absence of the site, accounts for the importance of the feature in the predictive model. To this end, we conducted an analysis of variance (ANOVA) test to examine the significance of the association between the presence of each nucleotide substitution and the growth-related dependent variable. We repeated the process for every base alteration for SNP sites that were found to be important by the gradient-boosting model across all cross-validation training data sets.

### Pangenome simulation.

To assess the impact of pangenome and population parameters on prediction, we simulated pangenome evolution under different combinations of the evolutionary constraints of gene gain/gene loss ratio and population size. We then used the pangenome data to predict the simulated growth yields, with increasing values for the penetrance of a causal allele. We simulated pangenomes using the *Simurg* package in R (66). To simulate different evolutionary scenarios and trait distributions, we first used a range of values for population size (*N* = 200, 400, and 600). We assumed an open pangenome model for bacterial pangenome evolution, in which the number of gene families increases with the addition of new genomes (67, 68). We therefore varied only the rate of gene acquisition (ν = 10^−7^, 10^−8^, or 10^−9^), which tunes the increase in pangenome size, to create different pangenome matrices. The gene acquisition values of 10^−7^, 10^−8^, and 10^−9^ correspond to accessory genome size of 77%, 23%, and 1% of the pangenome, respectively. We kept the rate of gene loss (γ) at a constant value of 10^−11^. This resulted in pangenomes of various sizes. We then randomly drew an accessory gene with a frequency in the range of 0.45 to 0.55 and labeled them causal. We assigned continuous growth values to strains using a distribution after fitting a normal curve with the mean values of μ and a standard deviation of σ to a realistic distribution, i.e., the distribution of growth yield values in the absence of drug treatment (Fig. S5A). For strains lacking the causal gene, growth values were randomly drawn from the baseline normal distribution with a mean value of μ, whereas for strains harboring the causal gene, random growth values were drawn from a normal distribution that had a mean value of μ + λσ, where λ corresponds to the selective advantage of the resistance gene. We screened a wide range of values for λ (0, 0.05, 0.1, 0.25, 0.5, 1, 1.5, 2, 5, and 10). We tuned and tested gradient-boosting regressors with 100 iterations and default parameters on the predictor and dependent data sets.

In order to compare simulation and actual results, we estimated the value for λ under antimicrobial treatments. To this end, we first fitted a bimodal mixed distribution to growth yield and doubling time distributions for each condition (Fig. S5A), as mentioned above, and then measured the difference between the means of normal distributions for susceptible and resistance subpopulations, as the experimental λ for the condition.

### Data availability.

The sequence data for the strains have been submitted to the European Nucleotide Archive (ENA) under the study accession number PRJEB23294.
