# Supplementary figures and images for: Machine Learning Prediction of Resistance to Subinhibitory Antimicrobial Concentrations from Escherichia coli Genomes
Source: mSystems. 2021 Aug 24;6(4):e00346-21. doi: 10.1128/mSystems.00346-21 (PMC8407197; doi:10.1128/mSystems.00346-21)

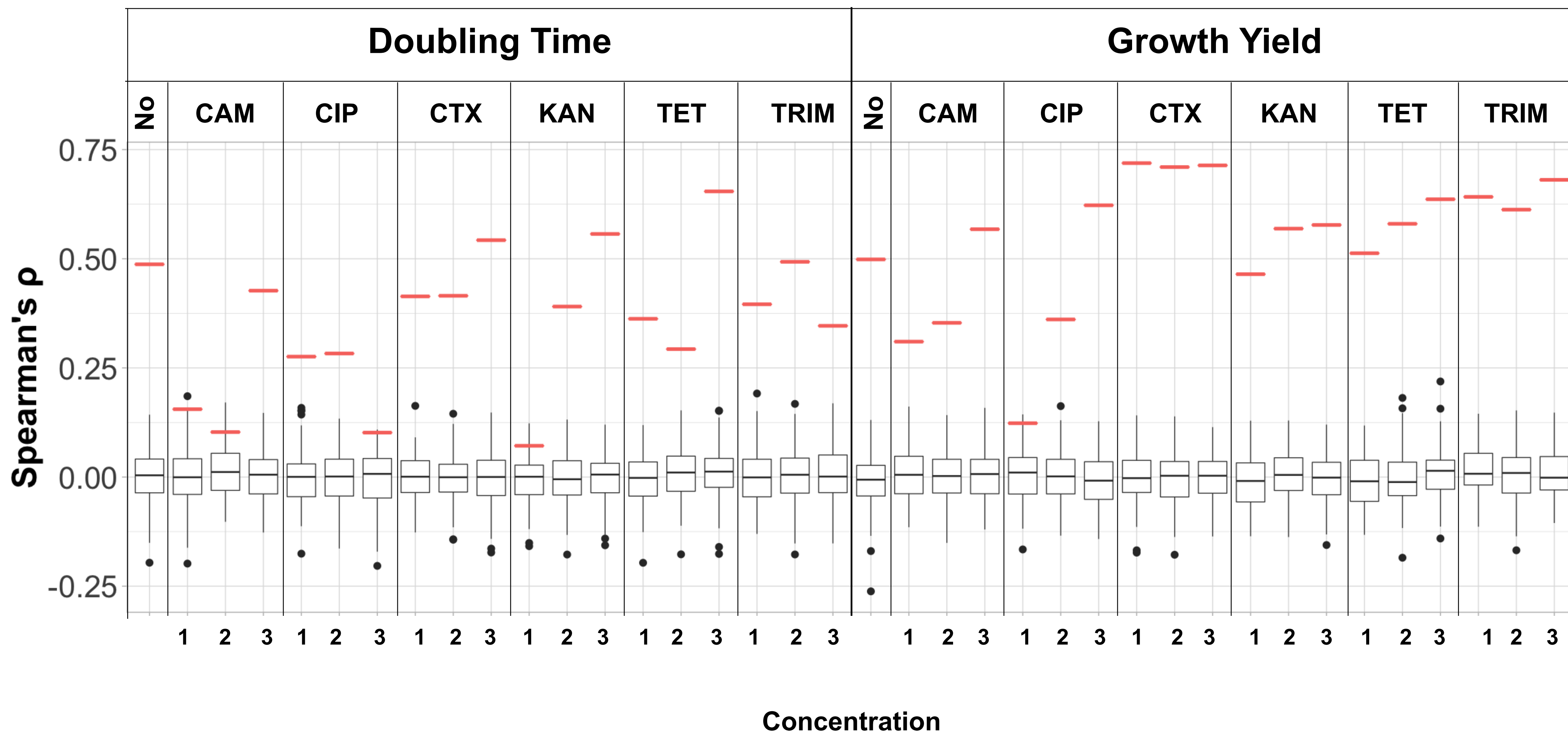

Supplement: FIG S1 [file msystems.00346-21-sf001.pdf]

## Doubling Time

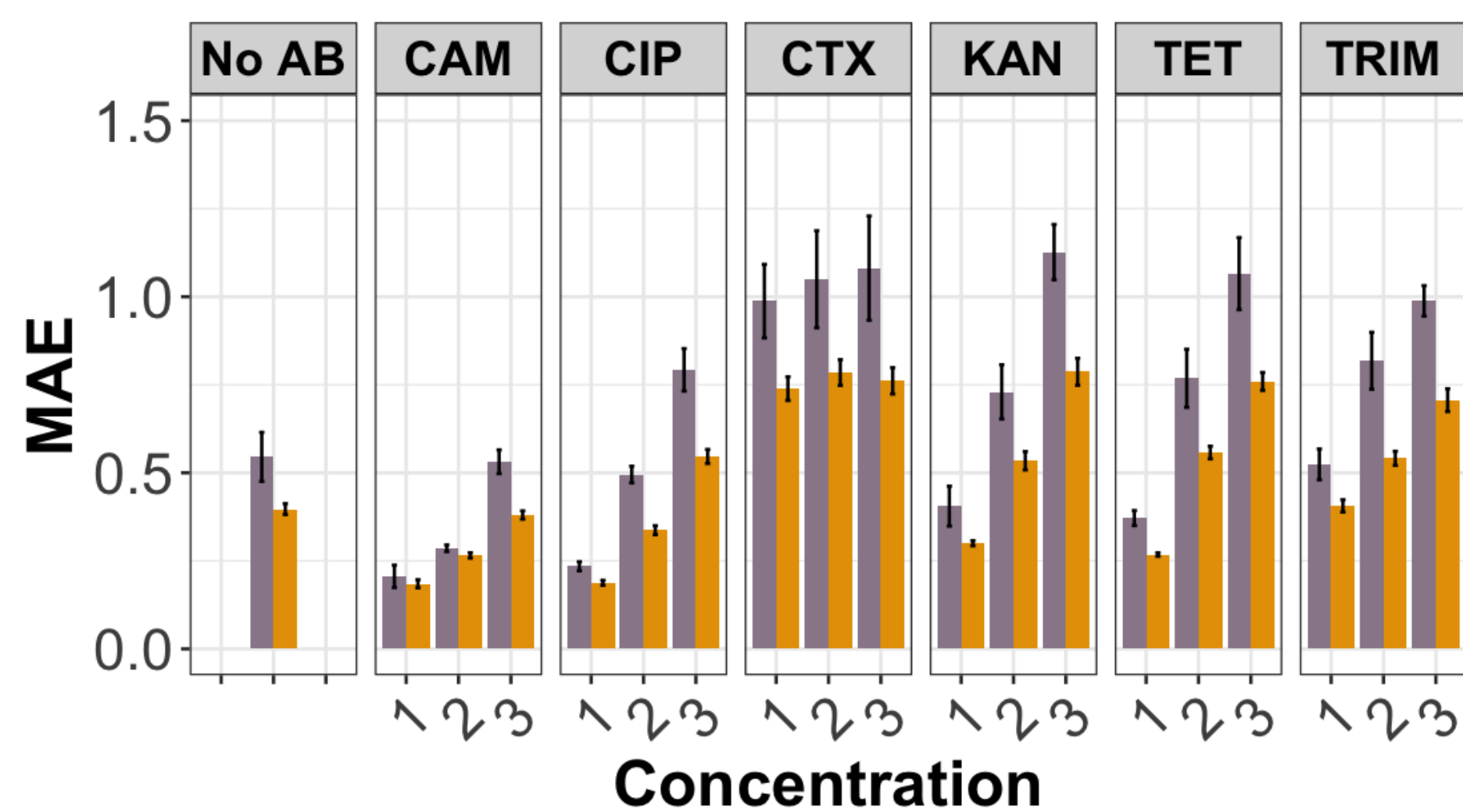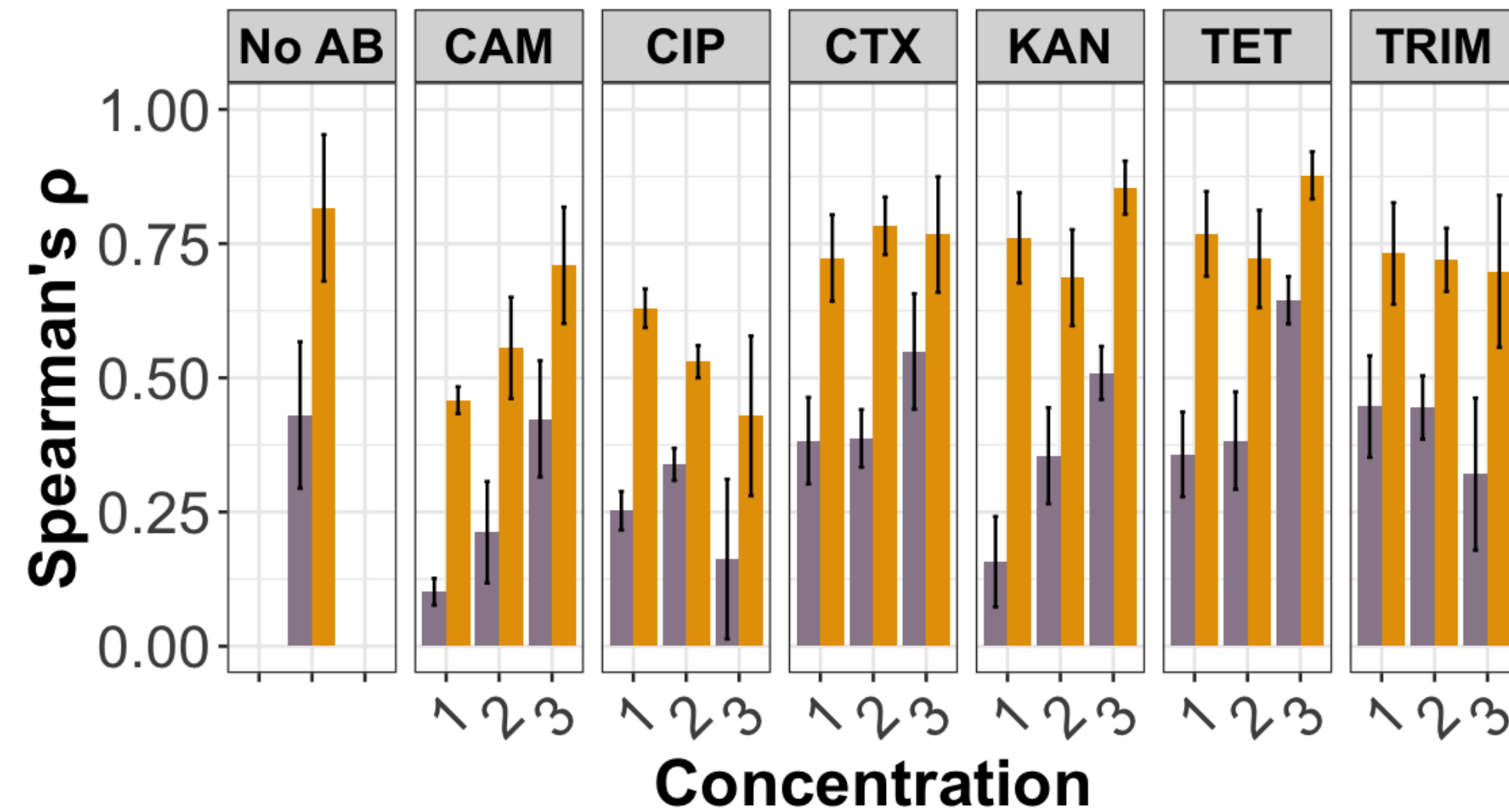

## Growth Yield

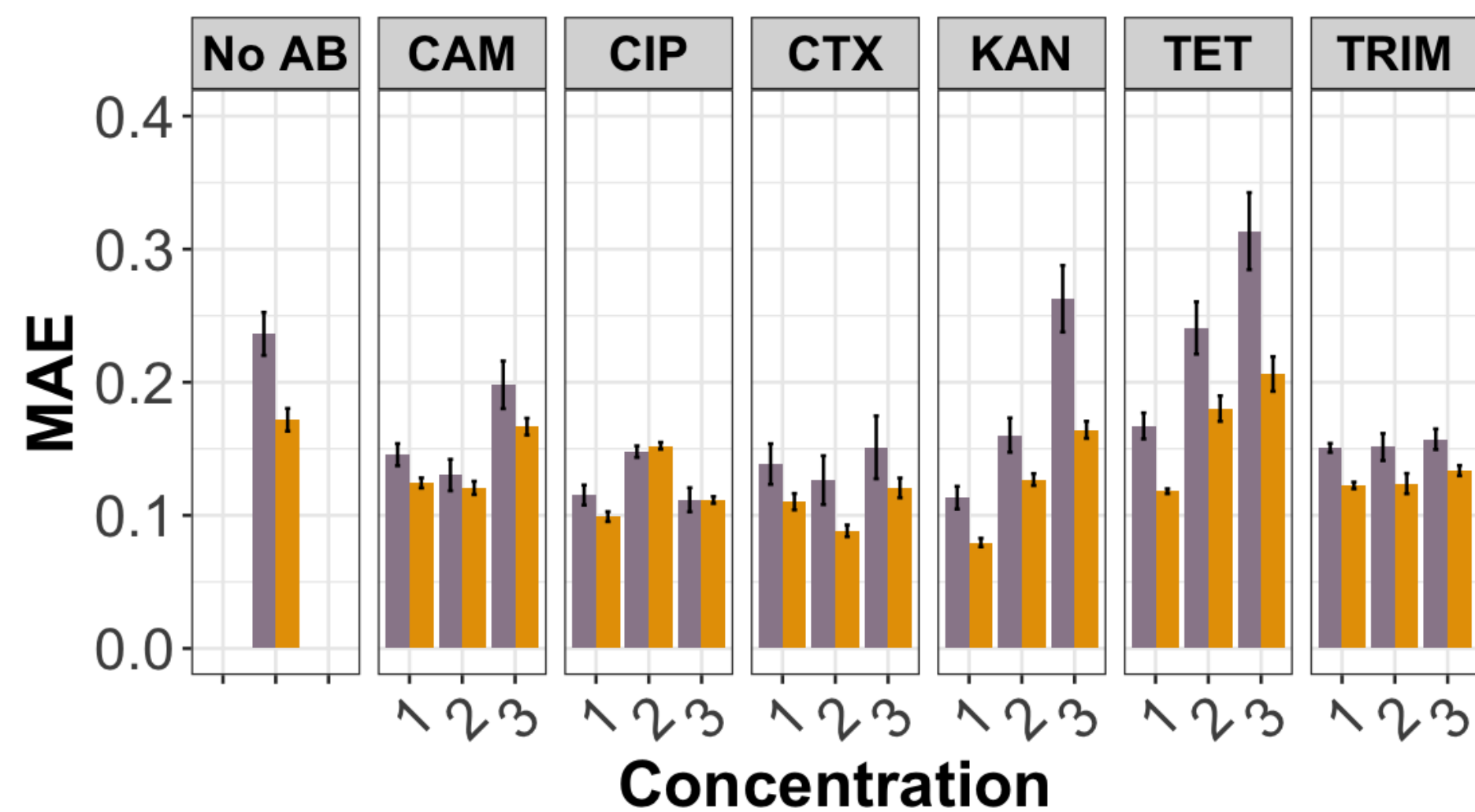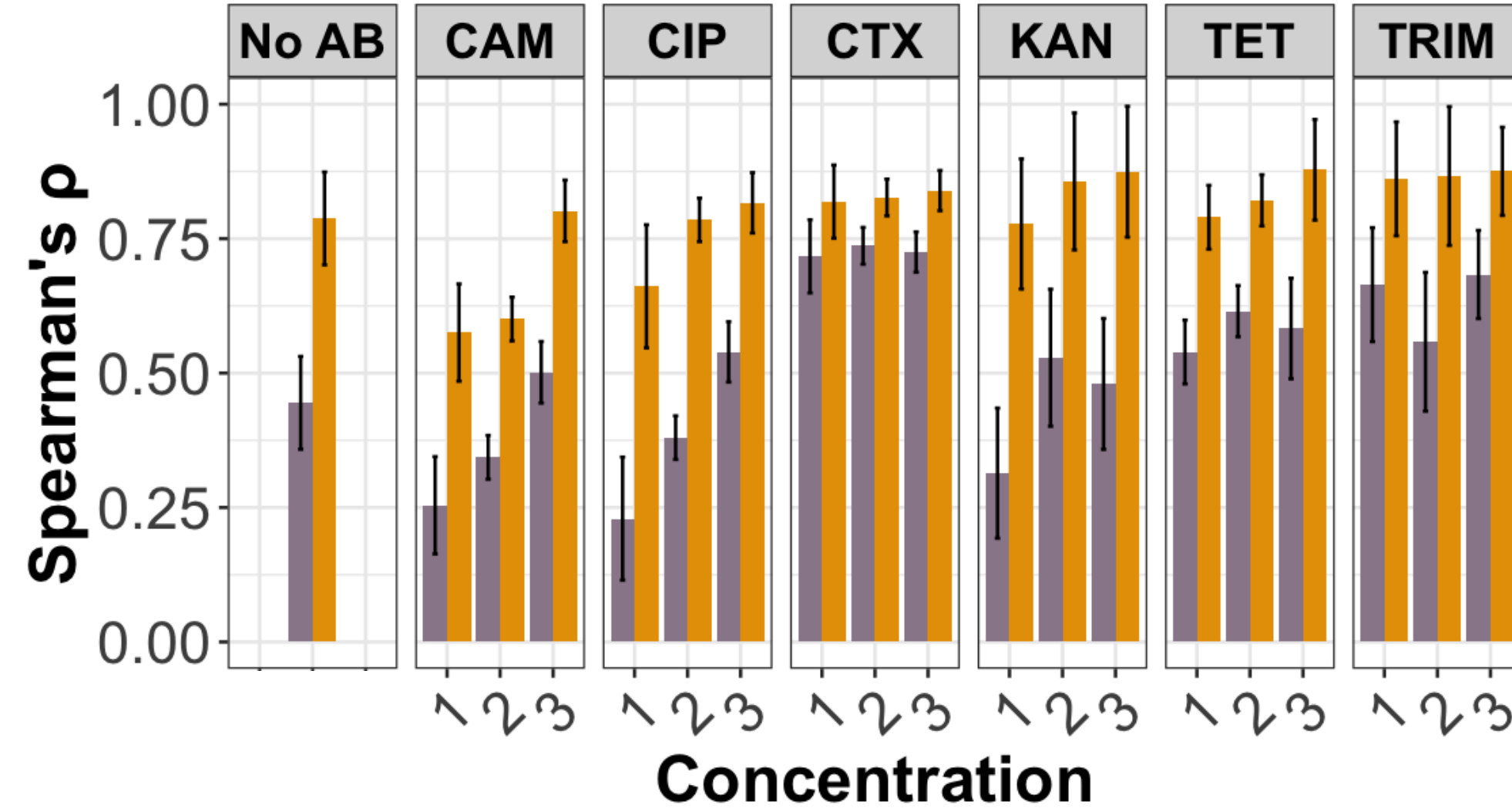

### Key

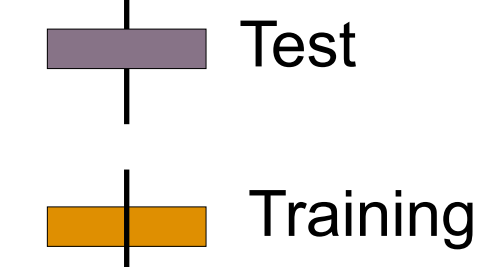

Supplement: FIG S2 [file msystems.00346-21-sf002.pdf]

A

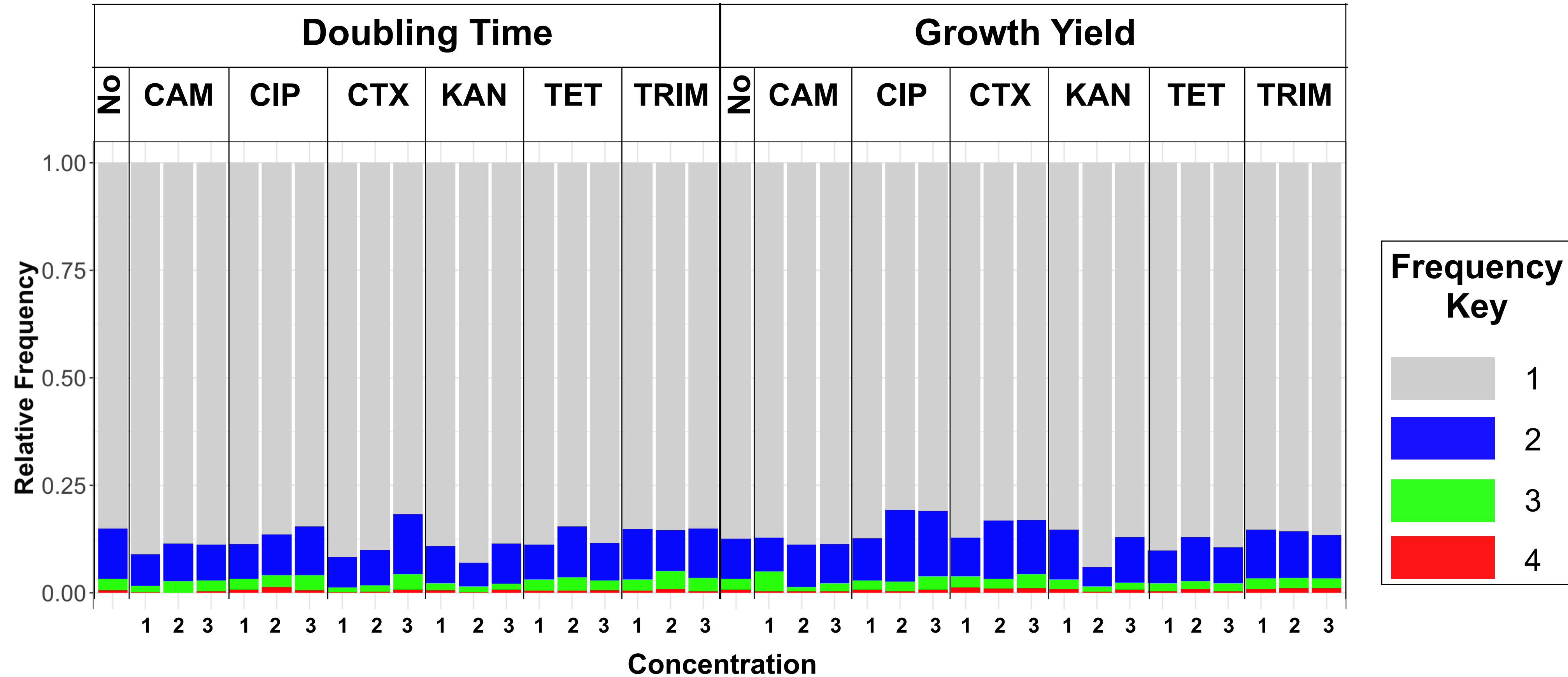

B

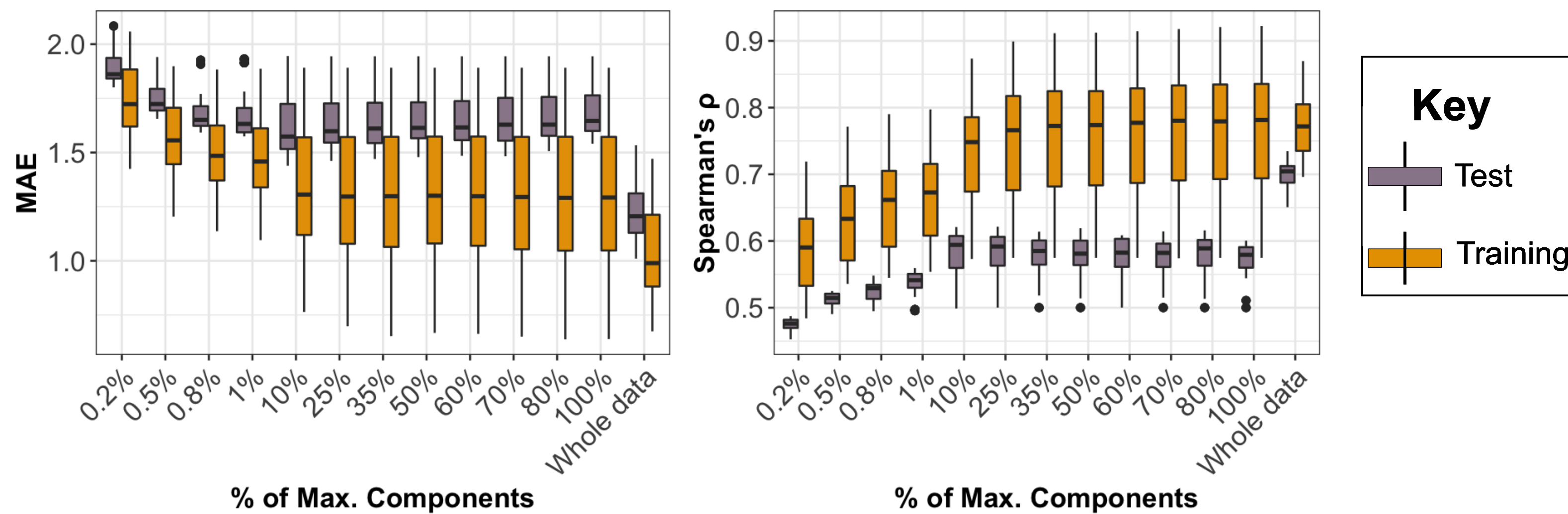

Supplement: FIG S3 [file msystems.00346-21-sf003.pdf]

# Doubling Time

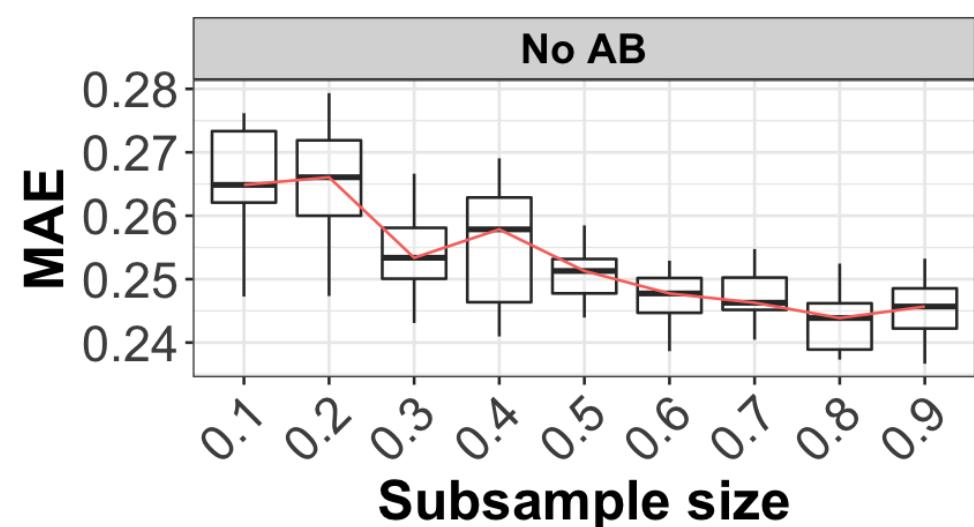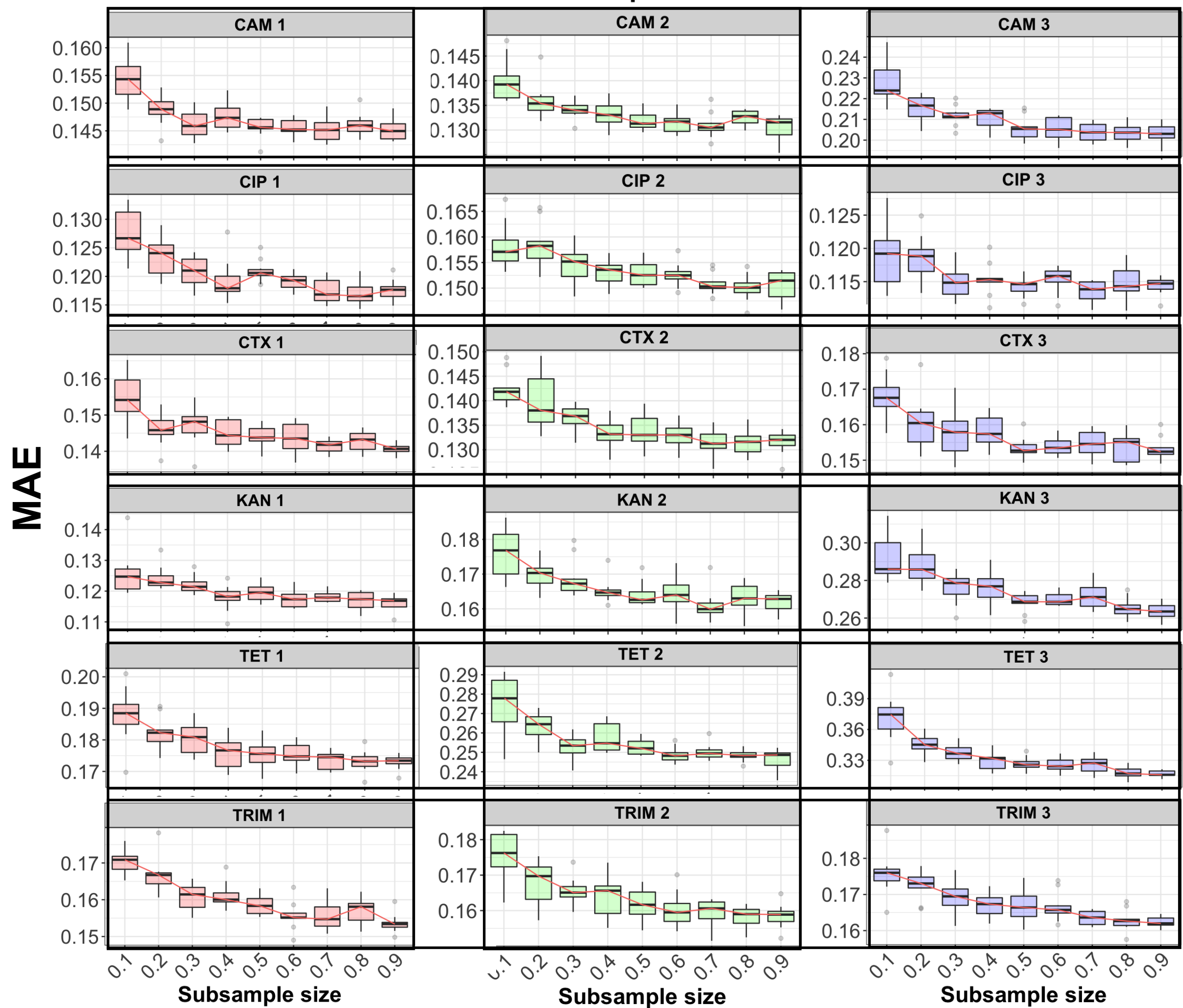

# Growth Yield

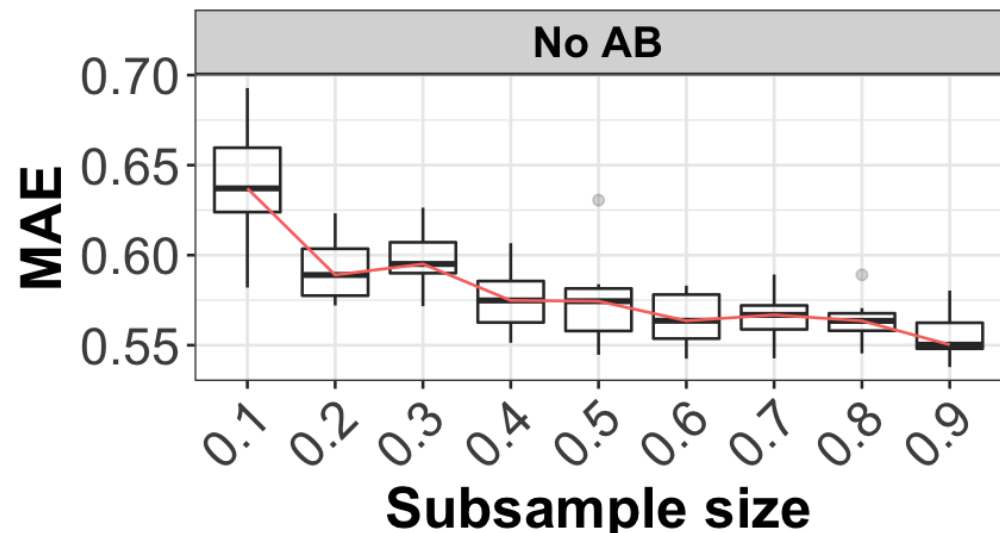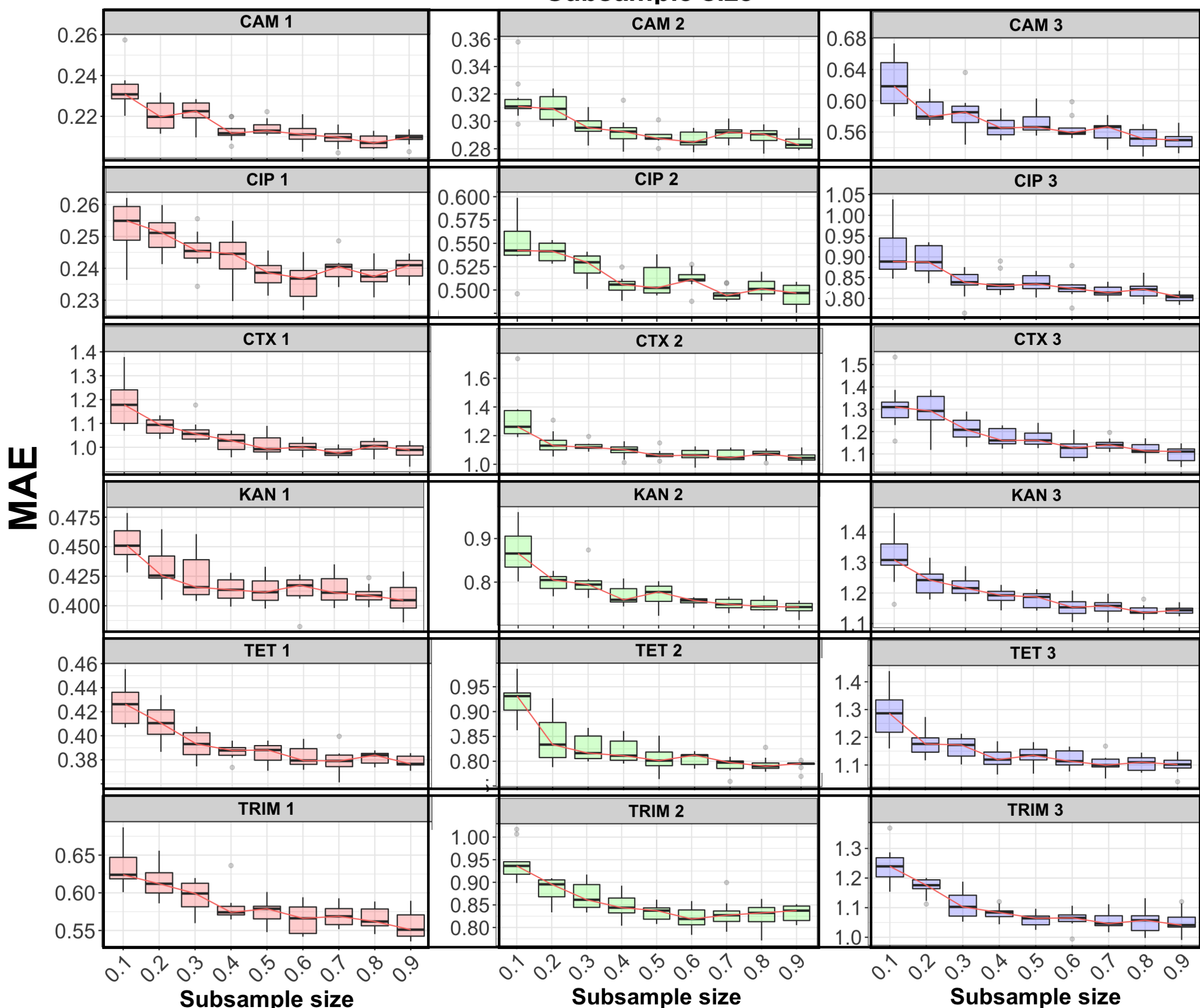

Supplement: FIG S4 [file msystems.00346-21-sf004.pdf]

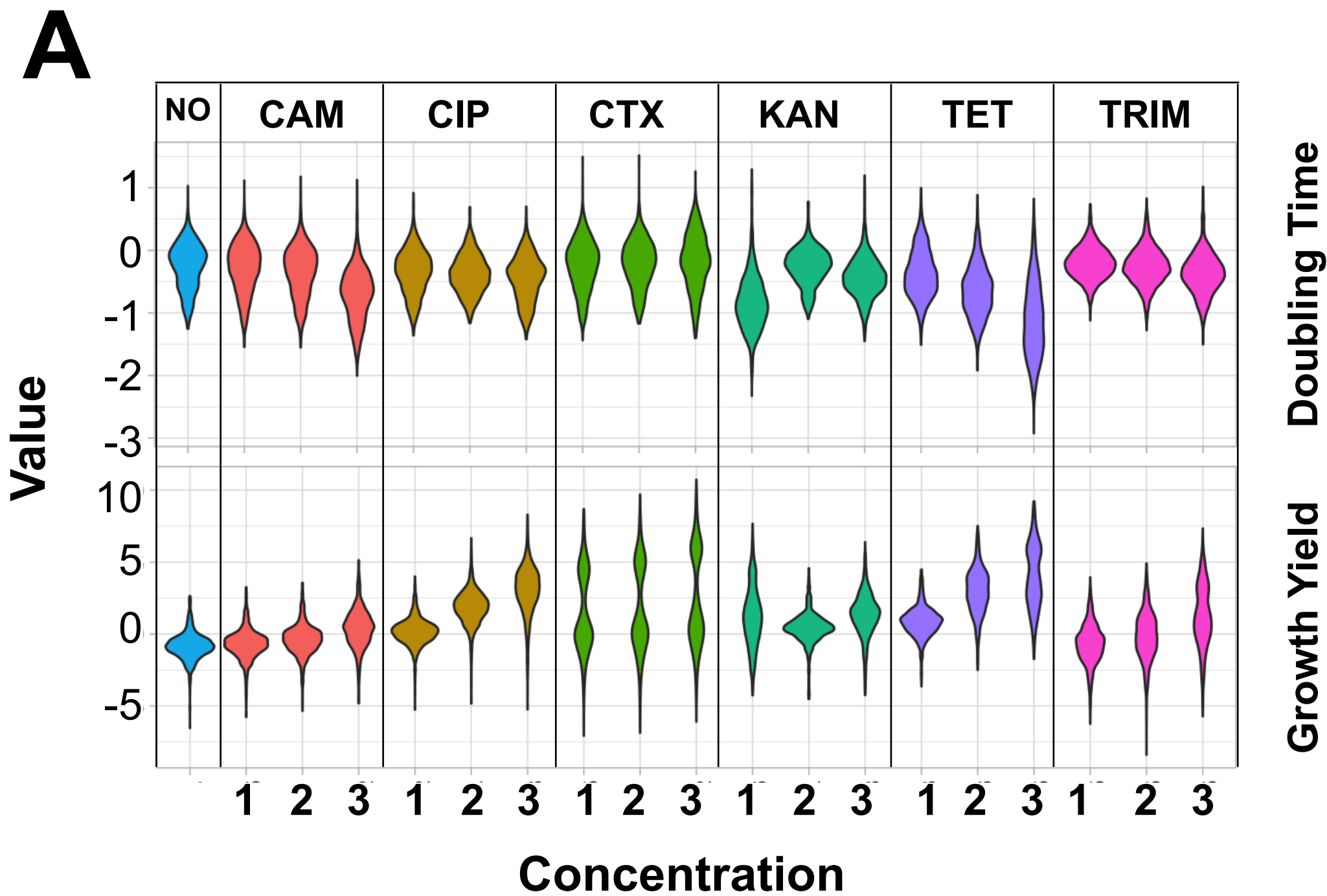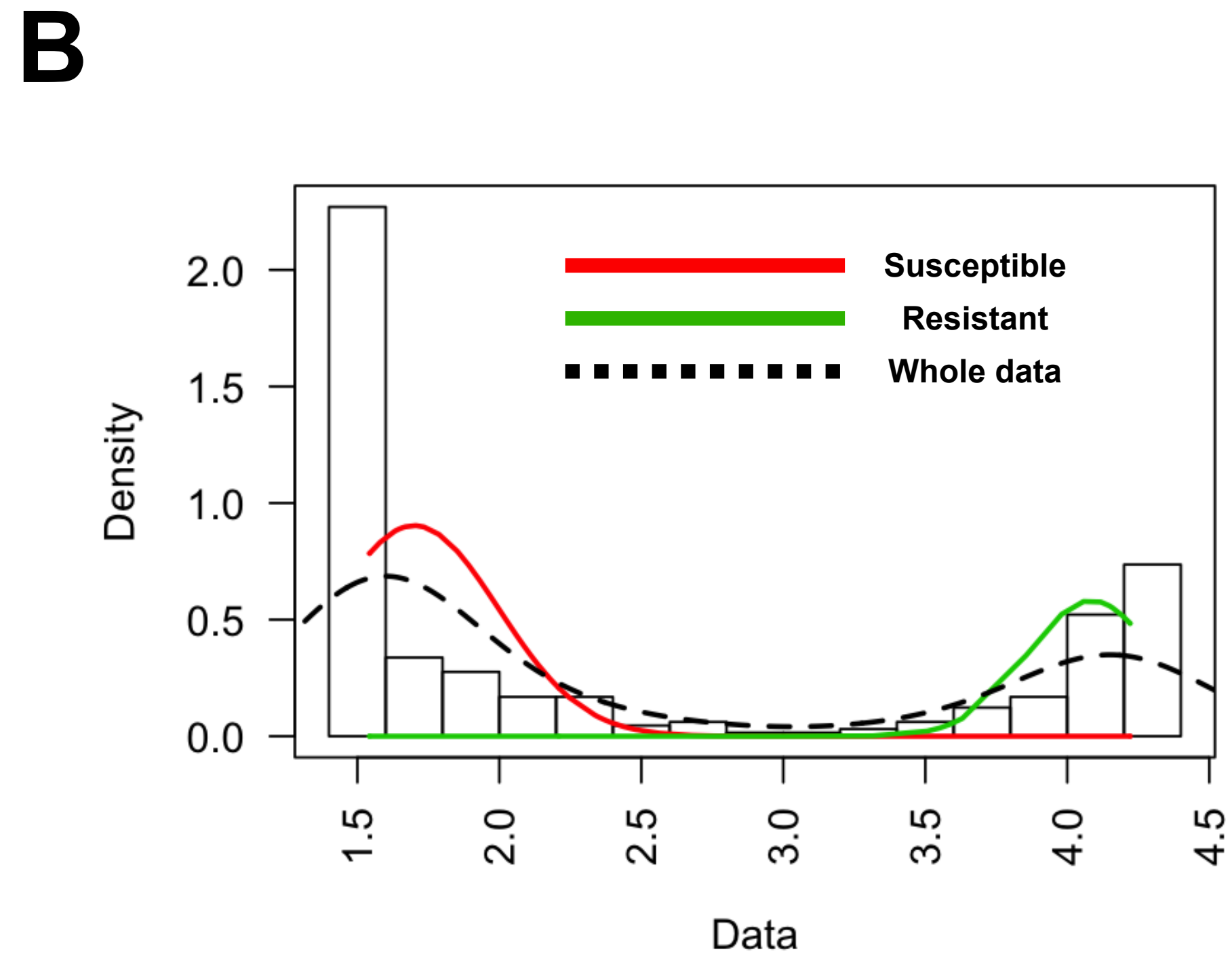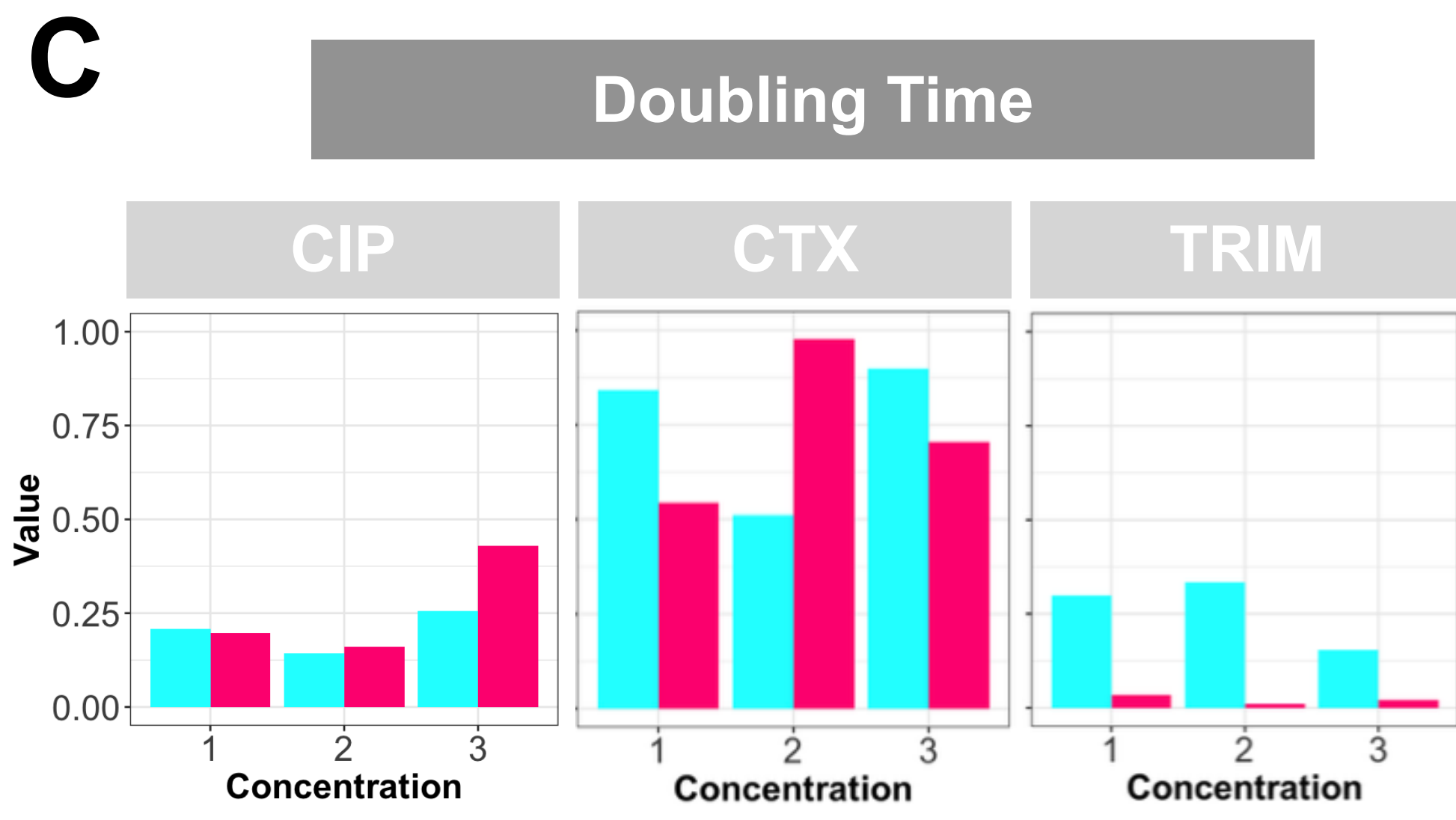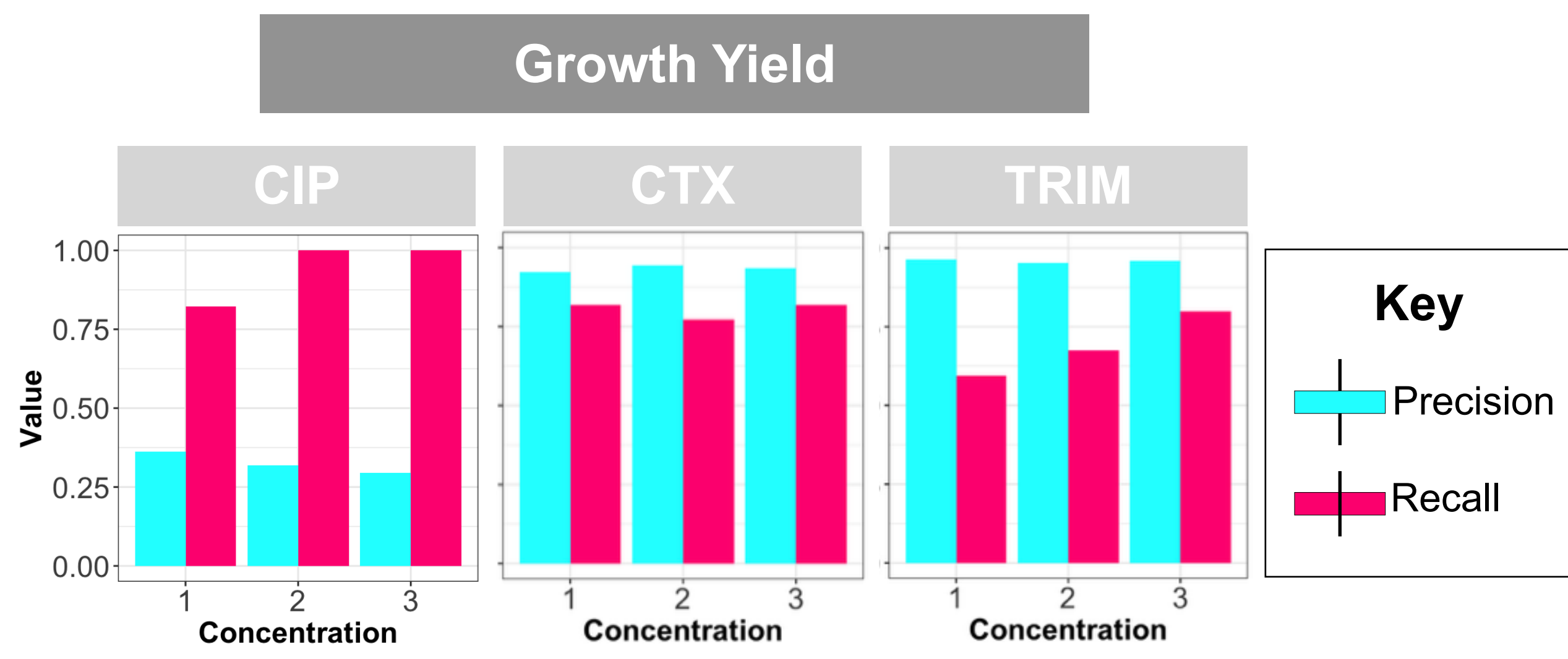

Supplement: FIG S5 [file msystems.00346-21-sf005.pdf]

SHAP

Concentration 1

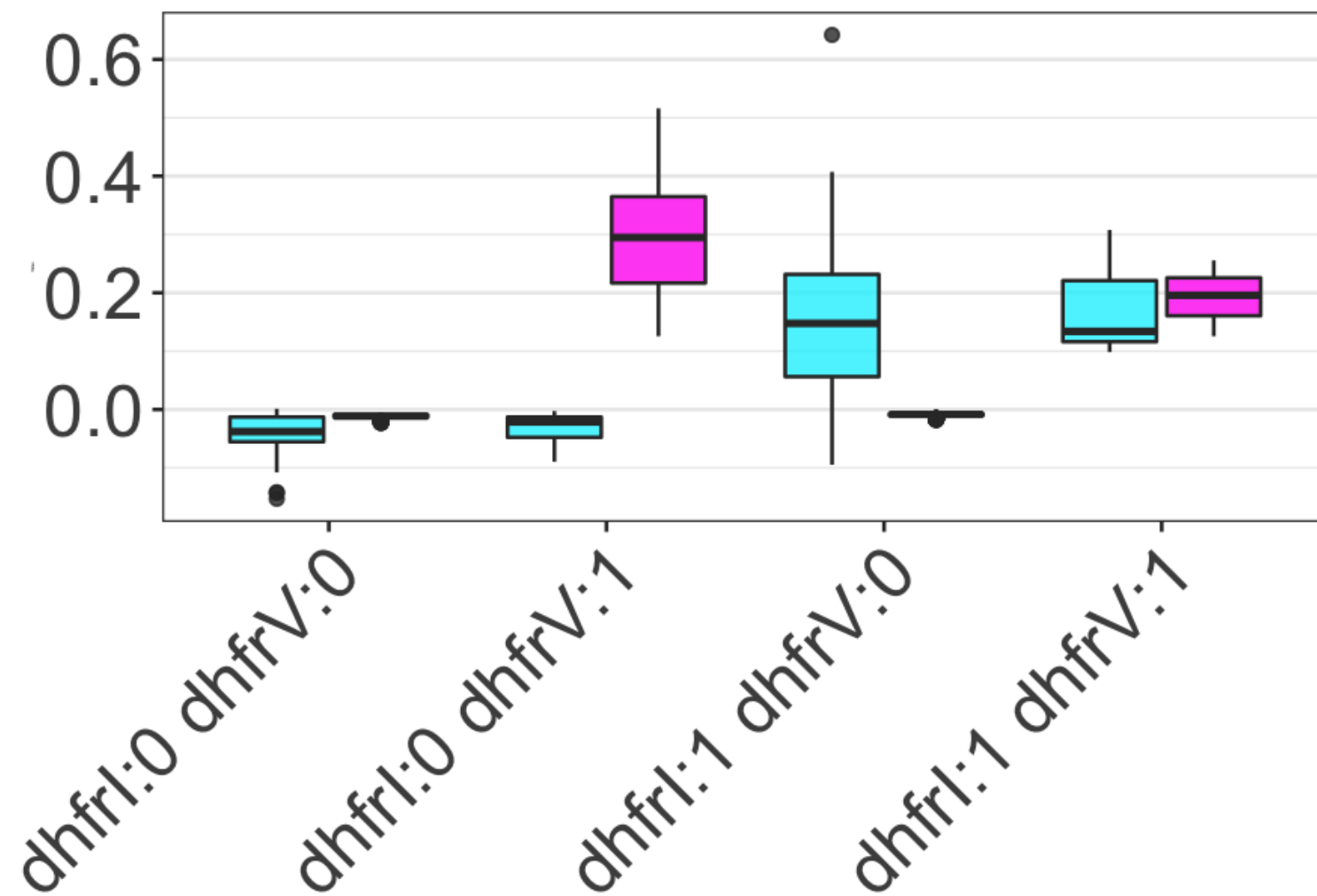

Concentration 2

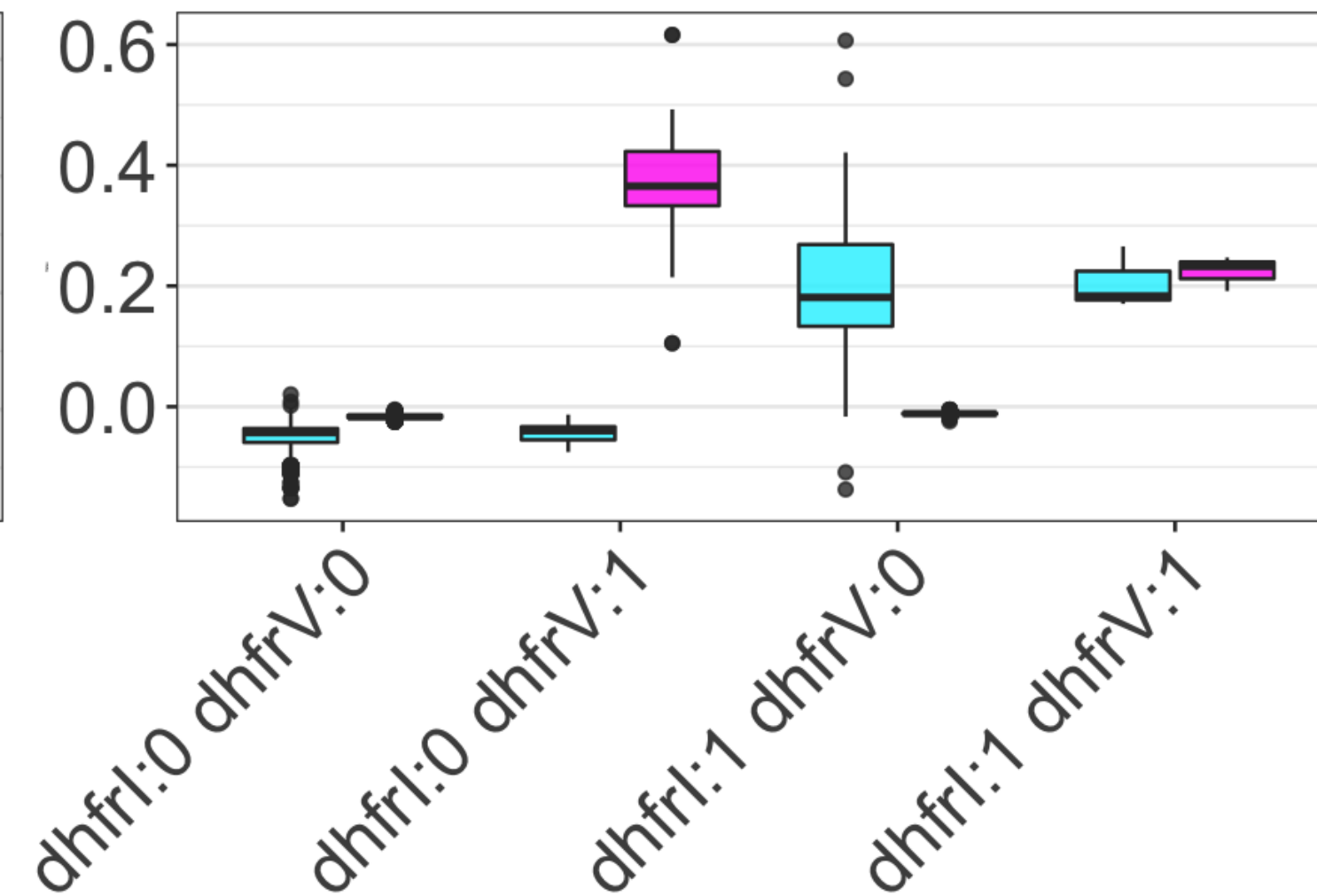

Concentration 3

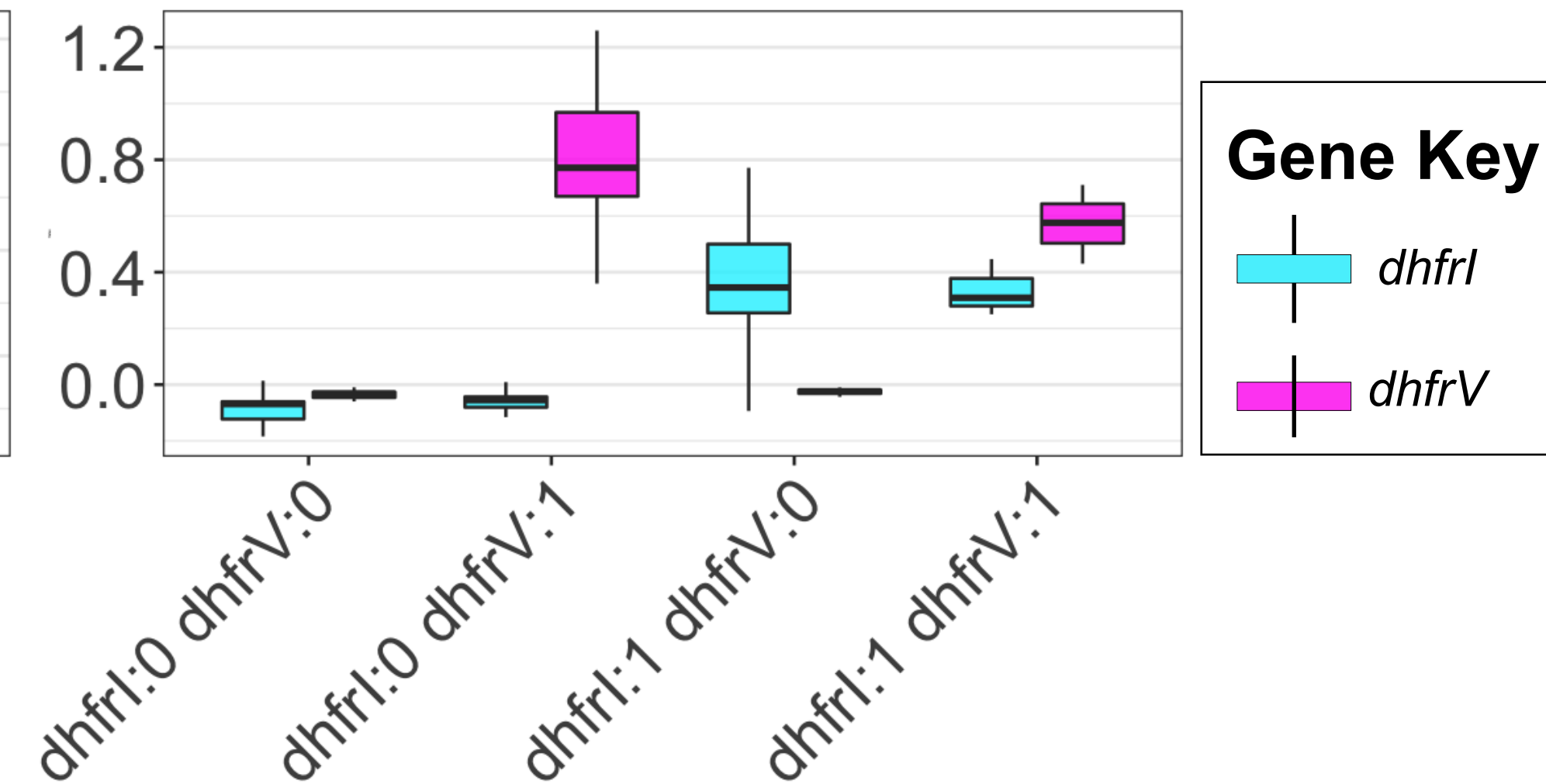

Gene Key

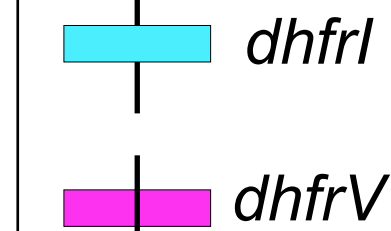

Supplement: FIG S6 [file msystems.00346-21-sf006.pdf]

A

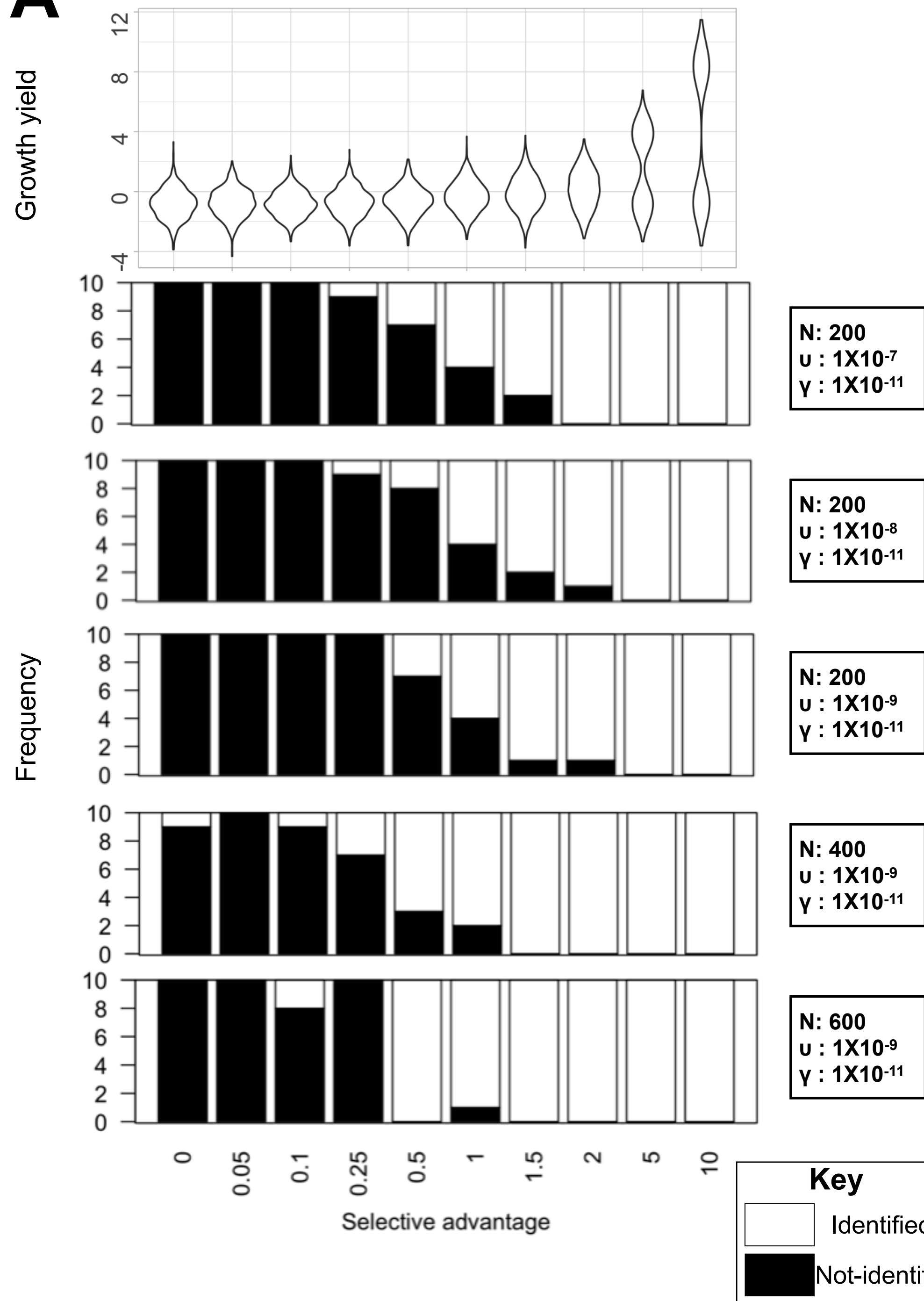

B

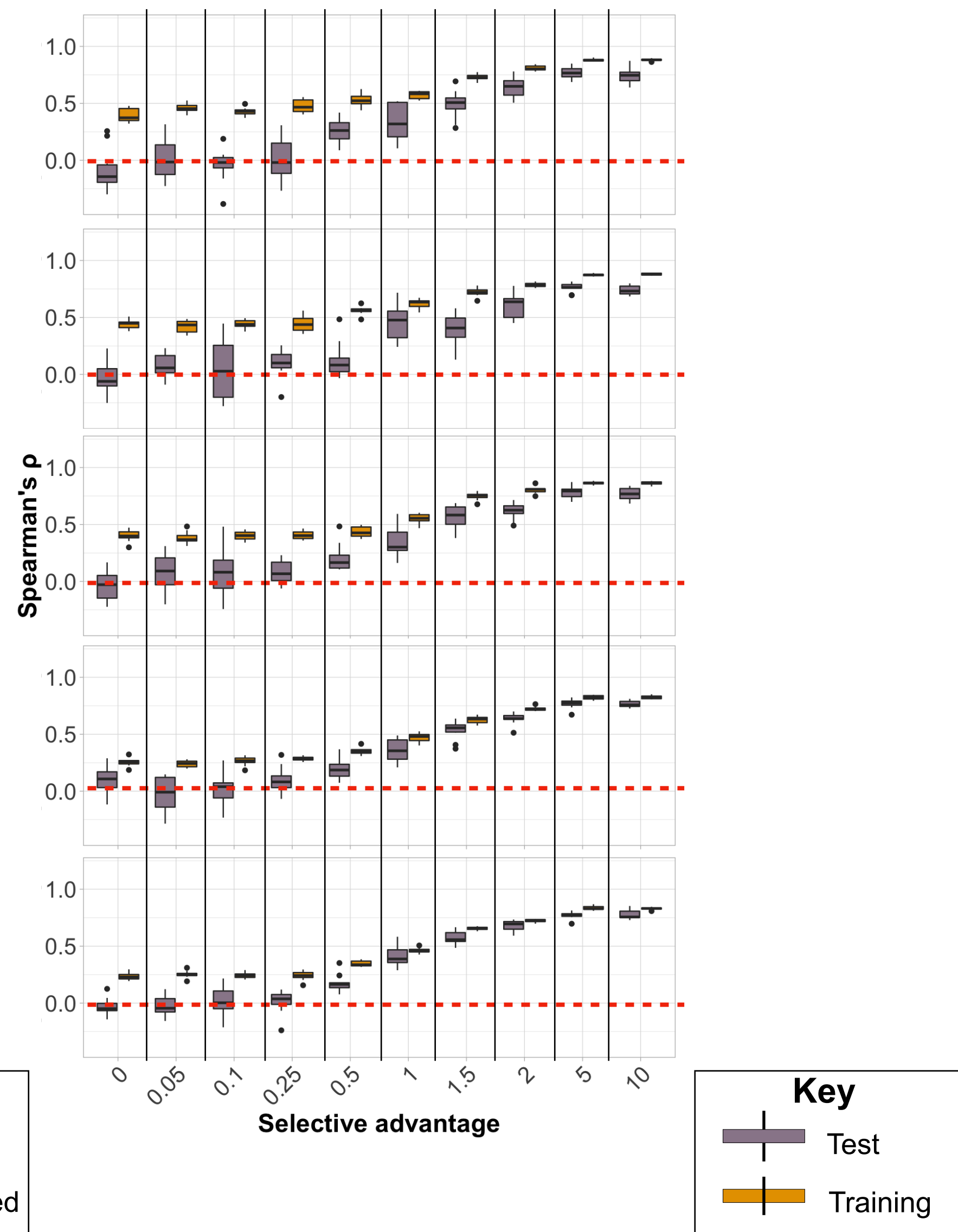

Supplement: FIG S7 [file msystems.00346-21-sf007.pdf]
